# Plasma Proteomics Reveals Persistent and Surgery-Responsive Molecular Signatures in Osteoarthritis Patients

**DOI:** 10.3390/ijms27062862

**Published:** 2026-03-21

**Authors:** Duygu Sari-Ak, Fatih Con, Melike Guvendi, Hayriye E. Yelkenci, Nazli Helvaci-Kurt, Alev Kural, Marcel Zamocky, Cemal Kural, Mustafa C. Beker

**Affiliations:** 1 Department of Medical Biology, Hamidiye International School of Medicine, University of Health Sciences, Istanbul 34668, Türkiye; 2 Clinic of Medical Biochemistry, Bakırkoy Dr. Sadi Konuk Training and Research Hospital, University of Health Sciences, Istanbul 34668, Türkiye; fatih.con@sbu.edu.tr (F.C.); alev.kural@sbu.edu.tr (A.K.); 3 Department of Endodontics, Institute of Health Sciences, Istanbul Medipol University, Istanbul 34668, Türkiye; melike.guvendi@std.medipol.edu.tr; 4 Regenerative and Restorative Medical Research Center (REMER), Research Institute for Health Sciences and Technologies (SABITA), Istanbul Medipol University, Istanbul 34668, Türkiye; heyelkenci@medipol.edu.tr; 5 Department of Medical Biochemistry, Hamidiye School of Medicine, University of Health Sciences, Istanbul 34668, Türkiye; 201002107@sbu.edu.tr; 6 Laboratory for Phylogenomic Ecology, Institute of Molecular Biology, Slovak Academy of Sciences, Dúbravská Cesta 21, SK-84551 Bratislava, Slovakia; marcel.zamocky@savba.sk; 7 Orthopaedics and Traumatology Clinic, Bakırkoy Dr. Sadi Konuk Training and Research Hospital, University of Health Sciences, Istanbul 34668, Türkiye; cemal.kural@sbu.edu.tr; 8 Department of Physiology, School of Medicine, Istanbul Medeniyet University, Istanbul 34668, Türkiye

**Keywords:** biomarkers, inflammation, osteoarthritis, proteomics, surgical intervention

## Abstract

Osteoarthritis (OA) represents a degenerative joint disease which advances through cartilage breakdown, synovial inflammation, and subchondral bone transformation until it causes persistent pain and mobility loss. The scientific community lacks complete knowledge about OA disease mechanisms and post-operative healing processes despite arthroplasty surgery providing effective symptom relief. This study investigated plasma proteomic changes in OA patients before and after arthroplasty. The cohort included eight OA patients undergoing knee or hip arthroplasty and ten age-, sex-, and body mass index-matched healthy controls. Plasma proteins were analyzed using liquid chromatography–tandem mass spectrometry following enzymatic digestion and depletion of high-abundance components. The bioinformatic analysis together with quantitative methods showed that OA patients experienced changes in inflammatory pathways, extracellular matrix remodeling, immune system regulation and coagulation processes. A total of 93 proteins were differentially abundant in the pre-operative comparison. Among these, 63 proteins were consistently up-regulated and 23 were consistently down-regulated across both pre- and post-operative time points. In addition, 20 proteins exhibited post-operative-specific changes. These findings highlight both persistent disease-associated alterations and transient proteomic shifts linked to post-operative recovery. Overall, this study identifies candidate plasma proteomic signatures associated with OA and surgical intervention, providing exploratory insights into disease monitoring and potential personalized therapeutic strategies.

## 1. Introduction

Osteoarthritis (OA) is a highly prevalent and disabling joint disorder that primarily affects older adults and substantially reduces quality of life. It is characterized by progressive articular cartilage degradation, subchondral bone remodeling, and synovial inflammation, collectively leading to chronic pain, stiffness, and impaired mobility. The prevalence of OA is closely associated with aging, most frequently affecting individuals over the age of 55 years [1]. With the global population aging rapidly, the incidence of OA is projected to increase substantially in the coming decades. Strikingly, in 2020, it was estimated that 595 million people worldwide were affected by OA, with projections indicating a 74.9% increase in knee OA cases by 2050. This increase in OA cases is further exacerbated by the growing rates of obesity, which is a significant risk factor for OA, particularly in the lower limbs [2,3].

The pathogenesis of OA is complex and involves a multifaceted interplay of mechanical, biological, and biochemical factors. Mechanical stress, often resulting from obesity, joint malalignment, or previous injury, is a major driver of disease initiation and progression. In parallel, genetic and epigenetic factors shape individual susceptibility and modulate disease trajectory. These risk factors lead to an overproduction of pro-inflammatory cytokines and catabolic enzymes, which accelerate cartilage degradation and joint destruction. The pain experienced by OA patients is also multifactorial, involving both nociceptive and neuropathic pathways, and is influenced by psychological and genetic factors. Despite the high prevalence and significant burden of OA, current treatment options are largely palliative, focusing on symptom management rather than disease modification. The absence of effective disease-modifying therapies underscores the urgent need for a deeper understanding of the molecular mechanisms driving OA [4,5,6].

In recent years, biomarker research has gained momentum as a strategy to improve early diagnosis, monitor disease progression, and evaluate therapeutic responses in OA. However, the marked molecular heterogeneity of OA and the complexity of its pathophysiology have substantially hindered the identification and validation of reliable and clinically translatable biomarkers [7,8,9,10,11]. Among the available approaches, proteomics has emerged as a particularly powerful tool, offering large-scale profiling of proteins and their post-translational modifications. Unlike genomics, the proteome dynamically reflects real-time cellular states under pathological conditions, thereby providing direct insight into active disease mechanisms. Advances in mass spectrometry-based proteomics have significantly enhanced our ability to identify disease-associated proteins and pathways, opening new avenues for biomarker discovery and the development of novel therapeutic targets [12,13,14,15].

In the current study, we conducted comprehensive proteomic profiling of plasma from OA patients before and after surgical intervention, together with healthy controls. This approach enabled us to capture both disease-related protein signatures and molecular shifts associated with post-operative recovery. By integrating mass spectrometry-based proteomics with bioinformatic analyses, we aimed to identify candidate biomarkers linked to inflammation, extracellular matrix remodeling, and metabolic regulation.

## 2. Results

### 2.1. Hematological and Biochemical Characteristics Across Study Groups

The hematological and biochemical parameters of controls, pre-operative (Pre-Op), and post-operative (Post-Op) OA patients are summarized in Table 1. The Pre-Op patients showed higher WBC counts and CRP levels than the controls, consistent with systemic inflammation, while the Post-Op values indicated further leukocytosis but a partial decline in CRP. The RBC, hemoglobin, and hematocrit decreased progressively from the controls to Post-Op patients, reflecting anemia exacerbated by surgery. Glucose levels were elevated in the OA patients, with further increases post-operatively, suggesting stress-induced hyperglycemia. Neutrophil counts rose and lymphocytes decreased after surgery, in line with acute inflammatory responses.

Renal and hepatic function markers remained largely within reference ranges, with minor decreases in creatinine and stable alanine aminotransferase and aspartate aminotransferase levels. Lipid profiles showed modest increases pre-operatively but returned closer to baseline after surgery. Electrolyte and albumin levels remained stable across groups. Clinically, all patients recovered well without post-operative complications.

### 2.2. Comparative Proteomic Analysis Between Pre-Op OA Patients and Healthy Controls

**Figure 1:**
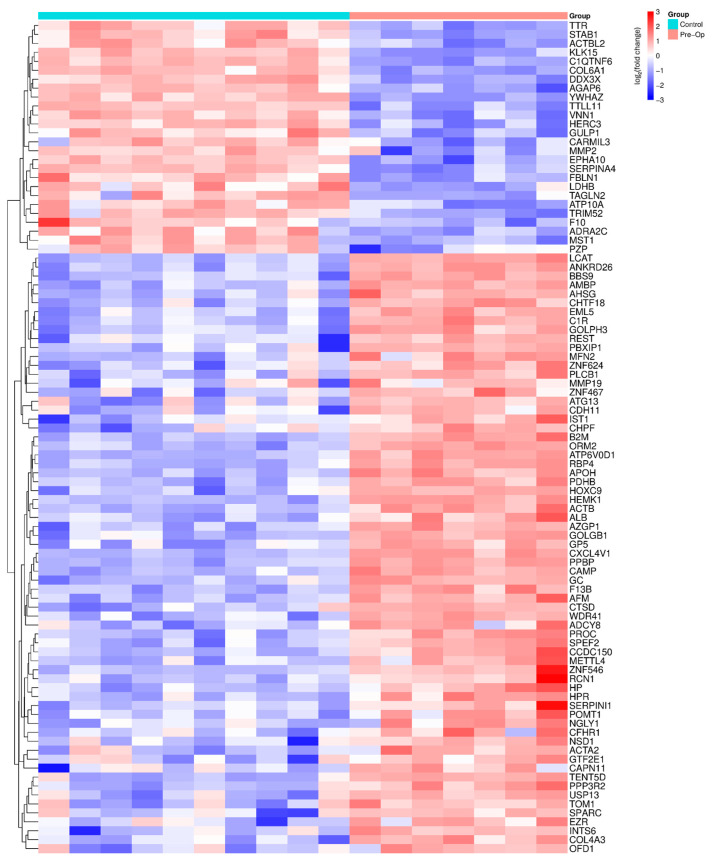
Heatmap of differentially abundant proteins between healthy controls and Pre-Op OA patients. Each cell shows the per-sample log_2_ fold-change relative to the control mean; rows and columns are hierarchically clustered. Colors denote relative abundance (blue = lower, red = higher; scale −3 to +3). Displayed proteins met the significance criteria *p* < 0.05 and |log_2_FC| > 1. The abbreviations of shown protein names are defined in Supplementary Table S1 and correspond to the Human Protein Atlas (https://www.proteinatlas.org, accessed on 17 March 2026).

Proteomic profiling of Pre-Op OA patients compared with controls (Figure 1) revealed significant molecular changes, identifying 93 differentially expressed proteins (*p* < 0.05, |log_2_FC| > 1). In total, approximately 67 proteins were significantly up-regulated, and 26 proteins were down-regulated, reflecting a dynamic reorganization of systemic protein expression.

Up-regulated proteins were primarily associated with hemostasis/coagulation (platelet activation/degranulation and fibrin-clot formation), innate immune/acute-phase responses (neutrophil degranulation, complement-like processes), and tissue remodeling (ECM organization/degradation and collagen turnover), alongside metabolic handling modules (scavenger-receptor-mediated heme/HDL remodeling), growth-factor regulation (IGF transport via IGFBPs), and cellular stress adaptations (autophagy, antioxidant activity, post-translational phosphorylation). Together, these enrichments indicate a pro-thromboinflammatory, remodeling-prone systemic state in pre-operative OA versus healthy controls, consistent with known OA biology involving platelet–coagulation–complement crosstalk, redox imbalance, and heightened matrix turnover, aligning with the pathophysiological framework of degenerative joint disease and its systemic spillover.

Down-regulated proteins were primarily associated with extracellular matrix organization/tissue remodeling and hemostasis/platelet activation, signaling, and aggregation. In Pre-Op OA versus controls, this downward shift is consistent with impaired matrix maintenance and dysregulated platelet–coagulation homeostasis, potentially reflecting consumption/redistribution or negative feedback within chronically activated thromboinflammatory pathways.

### 2.3. Comparative Proteomic Analysis Between Post-Op OA Patients and Controls

**Figure 2:**
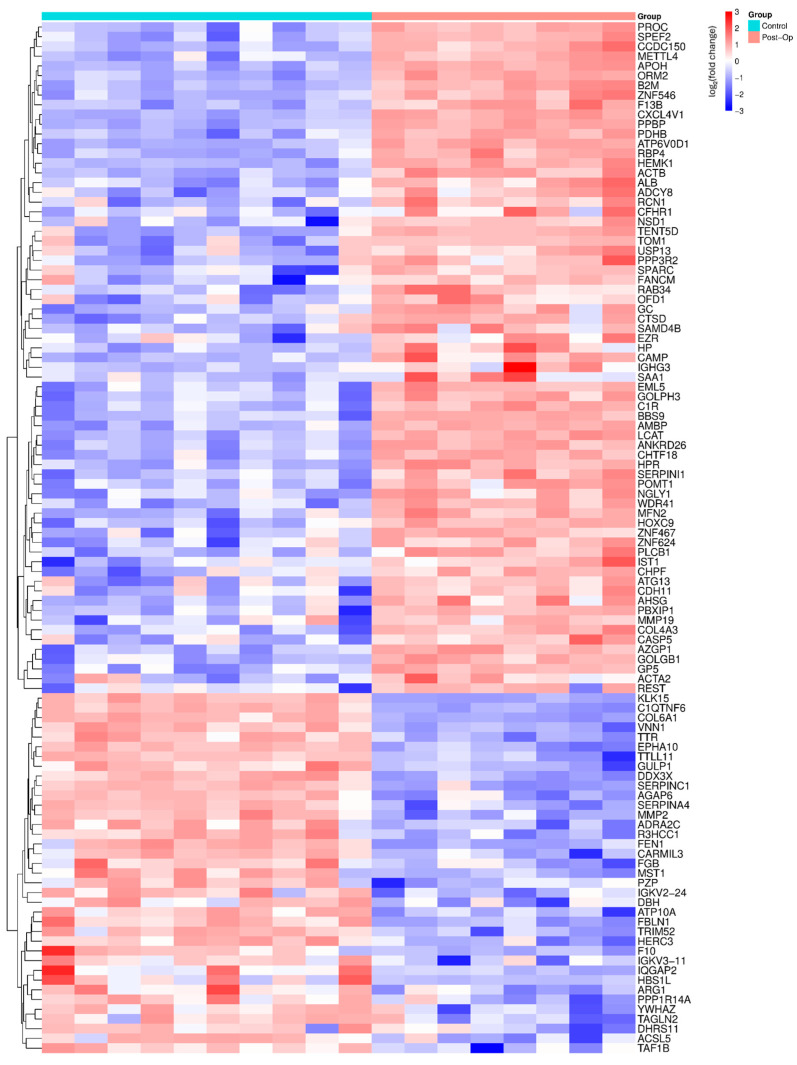
Heatmap of differentially abundant proteins between healthy controls and Post-Op OA patients. Each cell shows the per-sample log_2_ fold-change relative to the control mean; rows and columns are hierarchically clustered. Colors denote relative abundance (blue = lower, red = higher; scale −3 to +3). Displayed proteins satisfy *p* < 0.05 and |log_2_FC| > 1.

The analysis of the Post-Op proteomic dataset in comparison with healthy controls (Figure 2) demonstrated broad molecular remodeling, with 69 proteins up-regulated and 37 proteins down-regulated after surgery. Proteins that increased were predominantly associated with tissue regeneration, cellular stress adaptation, and immune regulation, pointing to active recovery processes. In contrast, proteins that decreased were mainly linked to inflammatory cascades, oxidative stress, and extracellular matrix turnover, consistent with a shift toward partial resolution of disease-driven catabolic activity. Importantly, a subset of proteins that had been markedly elevated pre-operatively declined toward baseline levels, further supporting the notion that surgical intervention modulates not only joint mechanics but also systemic molecular pathways.

Nevertheless, several regulators of immune and metabolic homeostasis remained dysregulated, indicating that the post-operative proteomic profile reflects an incomplete molecular recovery phase. These findings underscore the dual impact of surgery—mitigating pathological processes while simultaneously unveiling persistent alterations that could inform future biomarker development and adjuvant therapeutic strategies.

The proteins identified in Figure 1 and Figure 2, which show significant changes in Pre-Op and Post-Op conditions compared to the control group, are associated with specific biological pathways. These pathways and their corresponding proteins are detailed in Supplementary Tables S2 and S3, where the enrichment analysis of these proteins is provided. These tables help clarify the biological significance of the identified proteins and their involvement in various molecular pathways, offering a deeper understanding of the molecular mechanisms at play in OA.

### 2.4. Proteomic Shifts in OA: Differential Protein Expression Pre- and Post-Surgery and Their Implications for Recovery and Disease Monitoring

**Figure 3:**
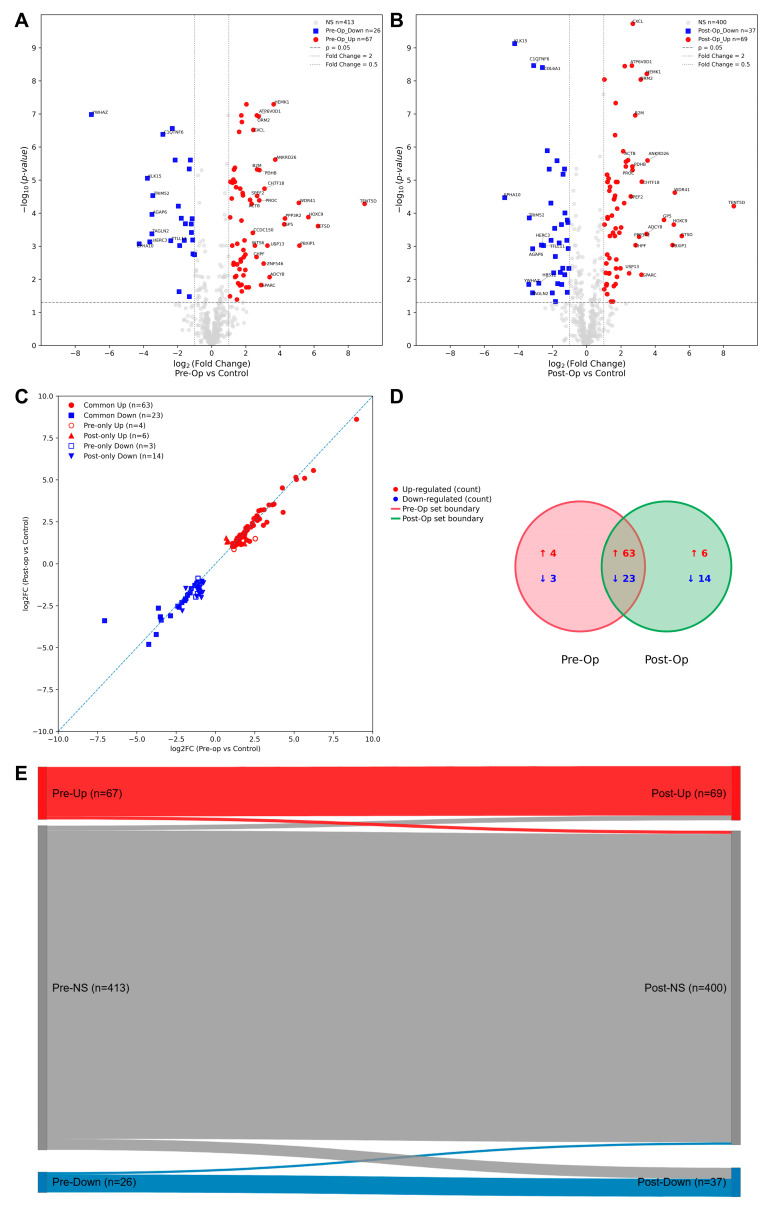
Integrated proteomic shifts in OA before and after surgery. (**A**,**B**) Volcano plots comparing the protein expression levels between OA patients and healthy controls. The left plot represents the comparison between Pre-Op OA patients and controls (**A**) while the right plot shows the comparison between Post-Op OA patients and controls (**B**). Each dot represents a protein (x-axis: log_2_ fold-change; y-axis: −log_10_ *p*-value). Significantly up-regulated proteins (red) and down-regulated proteins (blue) are highlighted at thresholds of *p* < 0.05 and |log_2_FC| > 1, and those showing at least a five-fold change in expression are additionally labeled. (**C**) Categorical scatter plot displaying log_2_FC values (Pre-Op vs. Control, x-axis; Post-Op vs. Control, y-axis) for all differentially abundant proteins. Shared (common) versus unique changes are indicated by distinct colors and marker shapes: common up (red circles), common down (blue squares), Pre-Op only up (open red circles), Post-Op only up (red triangles), Pre-Op only down (open blue squares), and Post-Op only down (blue triangles). (**D**) Venn diagrams showing the overlap of differentially abundant proteins. The circles represent the Pre-Op (left; red outline) and Post-Op (right; green outline) sets defined at *p* < 0.05 and |log_2_FC| > 1 versus controls. Within each region, counts are shown separately for direction: red “↑” = up-regulated, blue “↓” = down-regulated. For this cohort: Pre-Op only: ↑4, ↓3; shared (overlap): ↑63, ↓23; Post-Op only: ↑6, ↓14. (**E**) Sankey diagram showing transitions in differential abundance categories from Pre-Op to Post-Op (up, down, or not significant). Proteins were classified into three categories based on their differential abundance compared to healthy controls using thresholds of *p* < 0.05 and |log_2_FC| > 1: up-regulated (red), down-regulated (blue), or not significant (NS, grey). The widths of the flows represent the number of proteins transitioning between categories from the Pre-Op to Post-Op states. This visualization highlights proteins with persistent dysregulation (Pre-Up/Down → Post-Up/Down), those normalizing after surgery (Pre-Up/Down → Post-NS), and proteins exhibiting new alterations post-operatively (Pre-NS → Post-Up/Down). Together, these panels summarize the overall distribution, overlap, and dynamic transitions of proteomic alterations in OA patients before and after surgery, highlighting both persistent disease-related signatures and recovery-associated remodeling.

The overall distribution and overlap of differentially expressed proteins between Pre-Op and Post-Op OA patients are summarized in Figure 3. The Volcano plots illustrate the overall distribution of protein expression differences between the Pre-Op OA patients and controls (Figure 3A), as well as between the Post-Op OA patients and controls (Figure 3B). These plots highlight proteins with statistically significant changes, including those exhibiting at least a five-fold increase or decrease in abundance.

Among the up-regulated proteins, TENT5D, WDR41, and HOXC9 were the most prominent, along with ANKRD26, ADCY8, CTSD, GP5, PPP3R2, PBXIP1, HEMK1, USP13, CHTF18, SPARC, PDHB, PROC, SPEF2, B2M, ORM2, ATP6V0D1, ZNF546, CHPF, INTS6, CXCL4V1, CCDC150, and ACTB. Conversely, several proteins demonstrated significant down-regulation, including COL6A1 and C1QTNF6, as well as EPHA10, TTLL11, HBS1L, HERC3, AGAP6, TRIM52, KLK15, TAGLN2, and YWHAZ. These results identify the proteins with the most pronounced expression shifts in OA patients before and after surgery, defining the subset that constitutes the strongest molecular signature of disease and recovery.

A comparative analysis across time points revealed substantial overlap of differential signals (Figure 3C,D). Among the up-regulated proteins, 63 were shared between the pre- and post-operative states, with 4 unique to Pre-Op and 6 unique to Post-Op. For the down-regulated proteins, 23 were common in both comparisons, with 3 unique to Pre-Op and 14 unique to Post-Op. These patterns indicate that a core OA-related signature persists after surgery, while a subset of changes emerge post-operatively, consistent with ongoing systemic remodeling.

Within the pre-operative cohort, four proteins—AFM, CAPN11, GTF211, and INTS6—were significantly up-regulated versus controls (*p* < 0.05; |log_2_FC| > 1) but lost significance post-operatively (*p* ≥ 0.05), indicating attenuation of their differential abundance. Likewise, three proteins that were reduced at baseline—ACTBL2, LDHB, and STAB1—were no longer significant after surgery. Taken together, these “normalization-candidate” signals span transport/redox (AFM), proteolysis (CAPN11), transcription/chromatin-associated factors (GTF2E1, INTS6), cytoskeletal organization (ACTBL2), intermediary metabolism (LDHB), and scavenger-receptor pathways (STAB1), consistent with a partial dampening of pre-operative differences following surgery.

In contrast, a set of proteins that were non-significant at baseline became significantly altered after surgery (*p* < 0.05; |log_2_FC| > 1). Six proteins were up-regulated exclusively post-operatively—CASP5 (inflammatory caspase/innate immunity), FANCM (DNA damage response/replication-fork protection), IGHG3 (humoral immunity, IgG3), RAB34 (lysosome/vesicle trafficking), SAA1 (acute-phase/HDL remodeling), and SAMD4B (stress-responsive transcriptional control)—indicating post-surgical activation of innate and humoral pathways together with vesicle–lysosome dynamics. Conversely, fourteen proteins were down-regulated only after surgery, including ACSL5 and ARG1 (lipid and arginine metabolism), DBH and DHRS11 (oxidoreductase/catecholamine biosynthesis), FEN1 and TAF1B (DNA replication/Pol I transcription), FGB and SERPINC1 (hemostasis regulators; fibrinogen/antithrombin), IGKV2-24/IGKV3-11 (immunoglobulin components), IQGAP2 and PPP1R14A (cytoskeletal/contractile signaling), and R3HCC1/HBS1L (RNA/RNA-linked translation). Overall, the post-operative proteomic profile reflects an adaptive systemic response involving immune activation, controlled hemostatic adjustments, and metabolic reprogramming consistent with tissue repair and recovery.

Building on these observations, Figure 3E provides an integrated visualization using a Sankey diagram to illustrate the dynamic transitions in protein abundance between the pre- and post-operative states. The Sankey diagram shows the flow of proteins across three categories—up-regulated, down-regulated, and non-significant (NS)—highlighting persistent dysregulation (Pre-Op → Post-Op), normalization after surgery (Pre-Op → NS), and new alterations emerging only post-operatively (NS → Post-Op). Complementarily, the categorical scatter plot (Figure 3C) displays the log_2_FC values for each protein across both comparisons, distinguishing shared versus unique alterations using color and shape coding. This combined view facilitates the identification of proteins exhibiting sustained, resolving, or newly emergent changes in abundance following surgical intervention.

### 2.5. Pathway Analysis and Protein–Protein Interaction Network in OA Patients

**Figure 4:**
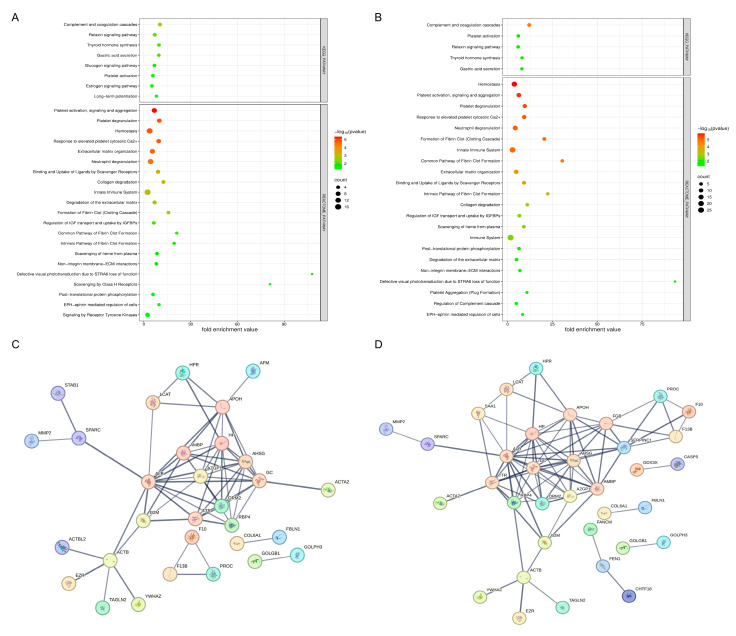
Pathway enrichment and protein–protein interaction networks in OA. (**A**,**B**) KEGG and Reactome pathway enrichment analyses of significantly altered proteins in Pre-Op (**A**) and Post-Op (**B**) OA patients compared with controls. Bubble plots display the most enriched pathways, with the x-axis representing the fold enrichment value, bubble size indicating the number of proteins involved, and color scale reflecting statistical significance (−log10 *p* value). Enriched pathways include complement and coagulation cascades, platelet activation and aggregation, innate immune responses, ECM remodeling, and lipid/HDL metabolism. (**C**,**D**) PPI networks constructed with STRING (confidence score ≥ 0.7) for Pre-Op (**C**) and Post-Op (**D**) differentially abundant proteins. Nodes represent proteins, edges indicate high-confidence interactions, and clusters highlight functionally interconnected modules.

Pathway analysis was conducted using the DAVID tool, focusing on the KEGG and Reactome pathways, to identify key signaling pathways involved in OA for the Pre-Op and Post-Op group (Figure 4A,B). This analysis revealed significant enrichment of several pathways, including those involved in immune responses, ECM interactions, and metabolic processes. Notable pathways included the regulation of the actin cytoskeleton, focal adhesion, and ECM–receptor interaction, all of which are critical in the progression of OA. Additionally, the Reactome pathway analysis underscored the importance of immune system function and inflammation in OA. Pathways related to platelet activation, complement and coagulation cascades, and inflammatory signaling were prominently featured. Other enriched pathways included lipid metabolism, scavenging of heme from plasma, and various post-translational modifications, emphasizing their roles in OA pathology.

To further elucidate the molecular mechanisms underlying OA, a PPI network for the Pre-Op and Post-Op OA groups was constructed using the STRING database, with a confidence score of 0.7 (Figure 4C,D). This network highlighted several central proteins that exhibited extensive interactions, playing crucial roles in OA progression. These proteins are likely to be involved in multiple pathways, such as inflammation (ORM2, AHSG, HP, SAA1, HPR), complement and coagulation cascades (APOH, FGB, F10, F13B, FBLN1, SERPINC1), extracellular matrix remodeling, antioxidant activity (FGB, AMBP), and metabolic regulation (HP, AMBP, APOH, B2M). Their high connectivity suggests they are key players in the molecular processes driving OA. These central proteins and their interactions represent potential therapeutic targets, as modulating their activity could influence several pathways simultaneously, offering a more strategic approach to OA treatment. The network also revealed clusters of proteins closely associated with specific biological processes, which may serve as functional groupings active in OA.

### 2.6. Focused Analysis of Highly Altered Proteins and Longitudinal Dynamics After Surgery

To focus on the strongest signals, proteins exhibiting at least a five-fold change relative to healthy controls (*p* < 0.05; |log_2_FC| ≥ 2.32) were examined. In total, 26 proteins met this criterion at both time points. An additional 7 proteins exceeded the fold-change threshold in only one comparison, and 1 protein (HBS1L) exceeded the fold-change threshold at both time points but reached statistical significance in only one comparison. Altogether, 34 proteins are summarized in Table 2, which provides fold-change values and statistical significance across Control, Pre-Op, and Post-Op groups. A corresponding heatmap representation is shown in Supplementary Figure S1 (log_2_FC scale, centered at 0), illustrating the overall clustering of these high-magnitude signals.

Proteins marked with a single asterisk (*) exceed the ≥5-fold change threshold in only one comparison (either Pre-Op vs. control or Post-Op vs. control). Non-marked proteins satisfy both the fold-change (≥5-fold) and statistical significance (*p* < 0.05) criteria at both time points. Proteins marked with a double asterisk (**) exceed the ≥5-fold threshold at both time points but reach statistical significance (*p* < 0.05) in only one comparison.

Several proteins, including ZNF546, USP13, and CCDC150, exhibited significant changes in the Pre-Op state but diminished post-surgery, with expression levels approaching those of healthy controls. Conversely, TTLL11, COL6A1, and HBS1L were expressed at lower levels in Pre-Op OA patients compared with controls and demonstrated a further significant five-fold decrease after surgery. TAGLN2 showed a ≥5-fold down-regulation exclusively in the Pre-Op comparison, whereas CHPF exhibited a ≥5-fold up-regulation only in the Post-Op comparison. These results emphasize the distinct proteomic shifts observed before and after surgical intervention and identify the proteins with the strongest differential expression across disease and recovery phases.

**Figure 5:**
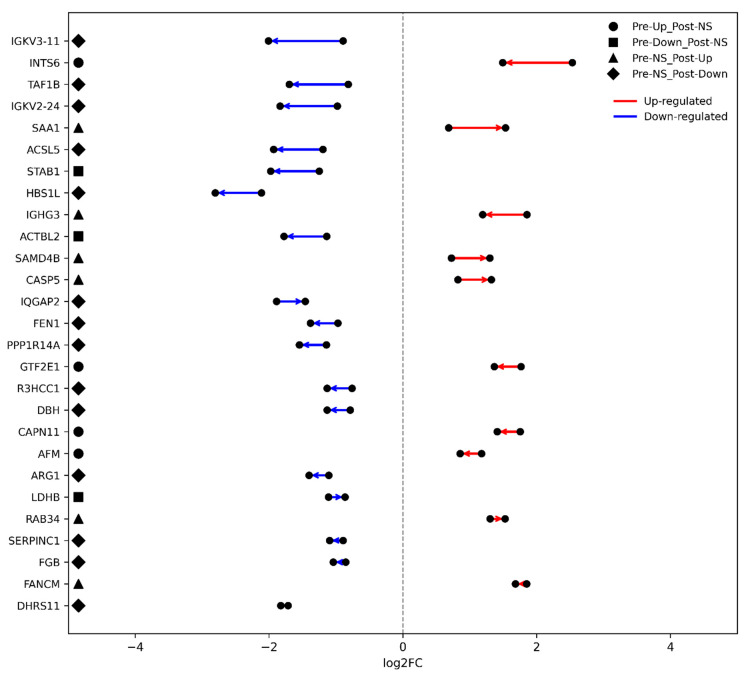
Slopegraphs illustrating longitudinal changes in significantly altered proteins across pre- and post-operative OA states. Proteins are shown in two categories: (i) significant in Pre-Op but non-significant in Post-Op (normalization candidates; 4 up-regulated, 3 down-regulated) and (ii) non-significant in Pre-Op but significant in Post-Op (newly emergent post-surgical changes; 6 up-regulated, 14 down-regulated). For each protein, a line connects its log_2_ fold-change values between Pre- and Post-Op, with the arrow indicating the transition direction (Pre → Post). Colors encode direction relative to controls (red = up-regulated, blue = down-regulated). Marker shapes denote category: circles (Pre-Up → Post-NS), squares (Pre-Down → Post-NS), triangles (Pre-NS → Post-Up), diamonds (Pre-NS → Post-Down). The protein list on the left includes all proteins across both categories.

To further explore the longitudinal direction and magnitude of proteomic alterations, Figure 5 illustrates slopegraphs focusing on two distinct protein subsets. The first group includes proteins that were significantly different in the Pre-Op state but lost significance after surgery—indicating normalization toward healthy control levels (four up-regulated and three down-regulated proteins). The second group comprises proteins that were non-significant pre-operatively but became significantly altered post-operatively, representing newly emergent post-surgical changes (6 up-regulated and 14 down-regulated proteins). Each line connects the log_2_FC values of a given protein between the Pre- and Post-Op states, with the arrow direction indicating the transition (Pre → Post). Color coding denotes up-regulated (red) and down-regulated (blue) proteins, facilitating visual interpretation of normalization versus emergence patterns relative to controls.

**Figure 6:**
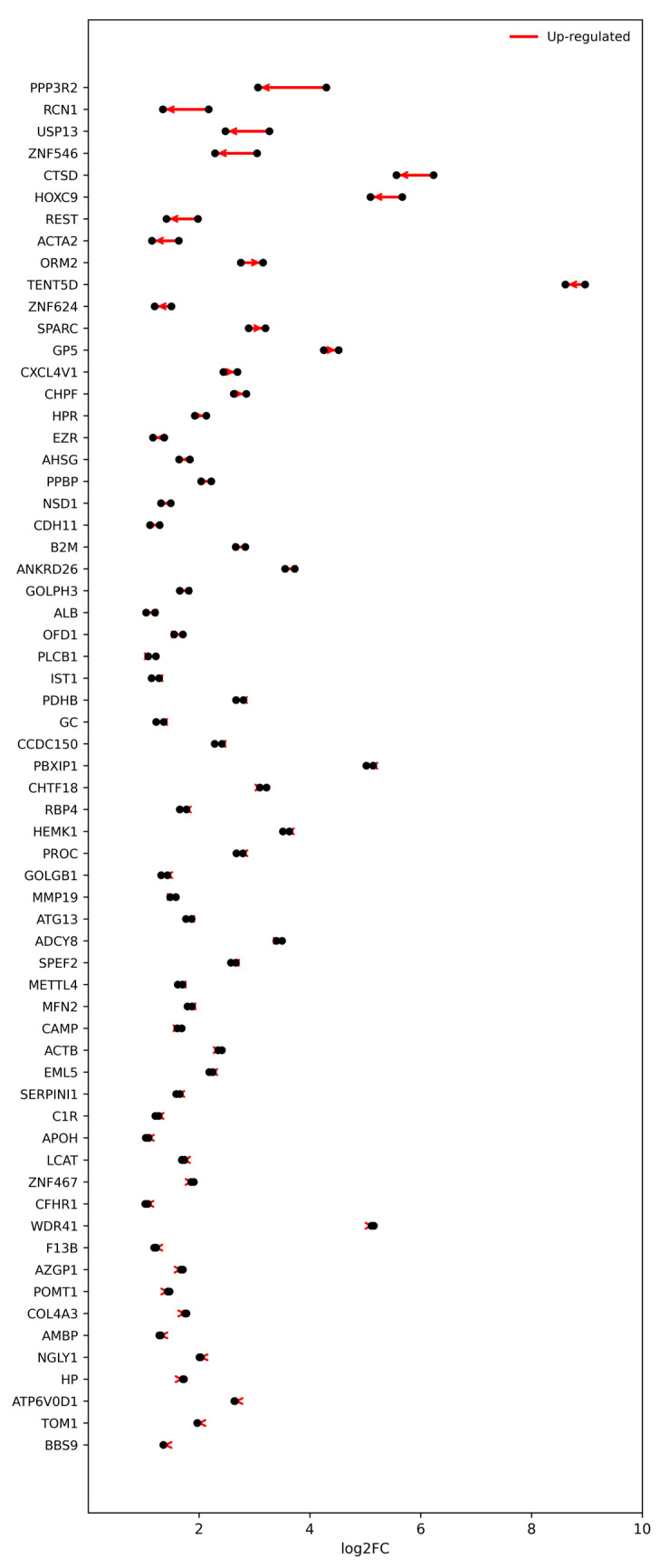
Proteins consistently up-regulated across pre- and post-operative OA states. Slopegraph depicting proteins that remained significantly up-regulated (*p* < 0.05; |log_2_FC| > 1) in both Pre-Op and Post-Op comparisons relative to healthy controls (*n* = 63). Each line connects the log_2_ fold-change (log_2_FC) values of the same protein between Pre- and Post-Op states, with arrows indicating the direction of change (Pre → Post). Red lines denote up-regulation relative to controls. Most proteins exhibit parallel or modestly declining trajectories, reflecting stable or partially attenuated overexpression following surgery. These consistently up-regulated proteins represent a core subset of OA-associated molecular alterations that persist beyond surgical intervention, highlighting processes related to inflammation, lipid transport, and extracellular matrix remodeling.

**Figure 7:**
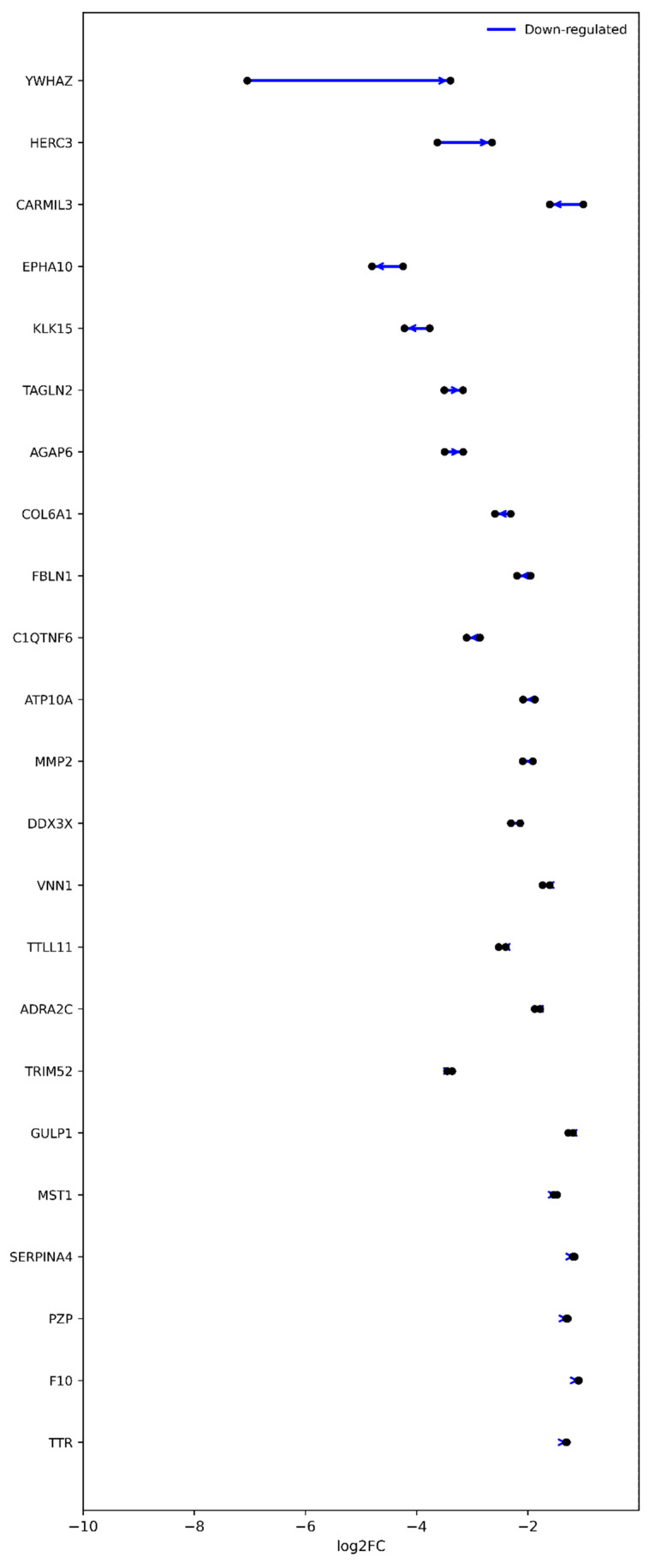
Proteins consistently down-regulated across pre- and post-operative OA states. Slopegraph illustrating proteins that remained significantly down-regulated (*p* < 0.05; |log_2_FC| > 1) in both Pre-Op and Post-Op comparisons relative to healthy controls (*n* = 23). Each line connects the log_2_ fold-change (log_2_FC) values for the same protein between Pre- and Post-Op states, with arrows indicating the direction of change (Pre → Post). Blue lines represent down-regulation relative to controls. Most proteins exhibit parallel or slightly intensified decreases, indicating persistent suppression of expression after surgery. These consistently down-regulated proteins reflect a stable component of the OA proteomic signature, associated with impaired extracellular matrix organization, cytoskeletal remodeling, and hemostatic regulation that remain uncorrected following surgical intervention.

To identify proteins showing persistent regulation across both surgical time points, we next focused on those maintaining statistical significance in both the Pre-Op and Post-Op comparisons. In total, 63 proteins remained consistently up-regulated, and 23 proteins remained consistently down-regulated relative to healthy controls. These persistent signatures represent stable molecular alterations that are unaffected by surgical intervention, suggesting chronic or baseline dysregulation associated with osteoarthritis pathophysiology. Proteins sustaining up-regulation across time points are visualized in Figure 6, whereas those maintaining down-regulation are shown in Figure 7. Together, these patterns describe a core proteomic signature of OA that persists beyond mechanical correction, potentially reflecting underlying inflammatory, metabolic, and extracellular matrix remodeling processes characteristic of the disease.

**Figure 8:**
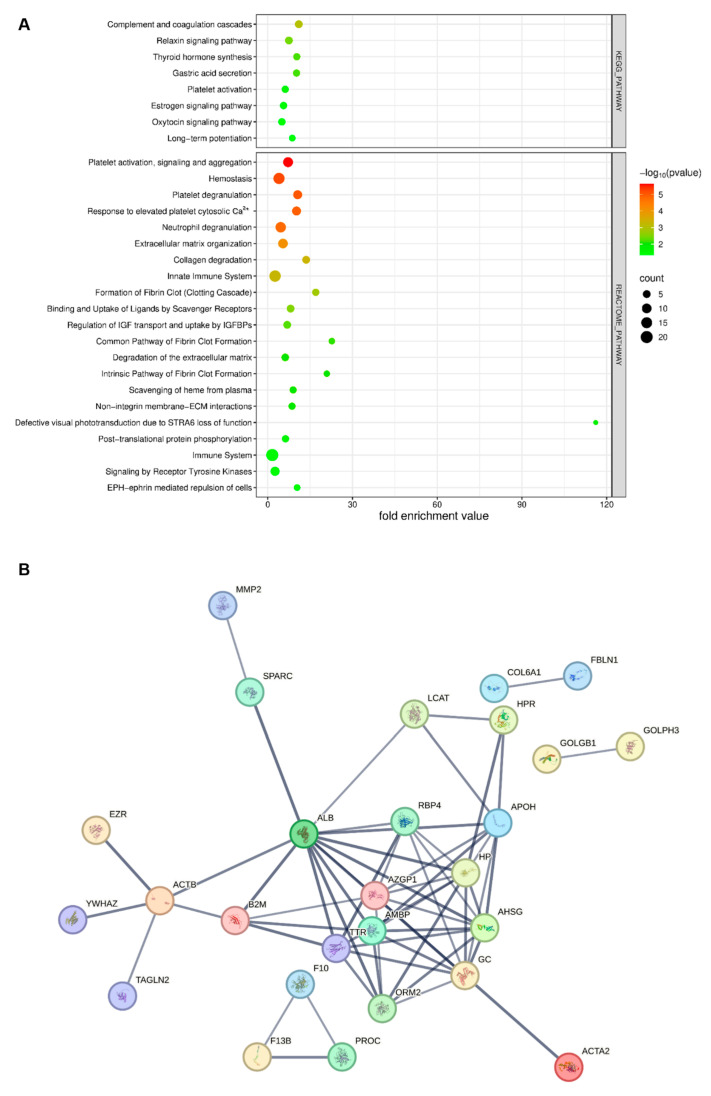
Pathway enrichment and PPI analyses of persistently altered proteins in OA. (**A**) KEGG and Reactome pathway enrichment analyses of proteins that remained significantly altered (*p* < 0.05; |log_2_FC| > 1) in both pre- and post-operative states compared with healthy controls (up-regulated *n* = 63; down-regulated *n* = 23). Bubble plots display the top enriched pathways, with the x-axis representing the fold enrichment value, bubble size indicating the number of proteins involved, and color scale denoting statistical significance (−log_10_ *p*-value). Up-regulated proteins were primarily enriched in complement and coagulation cascades, platelet activation, and acute-phase response pathways, whereas down-regulated proteins were enriched in extracellular matrix organization and cytoskeletal regulation. (**B**) STRING PPI network visualization (confidence score ≥ 0.7) of the persistently altered proteins. Nodes represent proteins, and edges indicate high-confidence interactions.

To further characterize the biological context of proteins that remained significantly altered at both time points, KEGG/Reactome pathway enrichment and STRING-based PPI analyses were performed (Figure 8). Distinct enrichment profiles were observed for the persistently up- and down-regulated protein subsets. The up-regulated group (*n* = 63) showed strong associations with complement and coagulation cascades, platelet activation, lipid metabolism, and acute-phase response pathways, consistent with sustained pro-inflammatory and pro-thrombotic signaling in OA. In contrast, the down-regulated group (*n* = 23) was mainly enriched in extracellular matrix organization, cytoskeletal structure, and cell–matrix interaction pathways, reflecting persistent suppression of structural maintenance mechanisms. STRING network visualization further highlighted interconnected clusters within both subsets, suggesting coordinated dysregulation of immune, metabolic, and matrix remodeling processes that remain active despite surgical intervention.

### 2.7. Experimental Validation

To validate the proteomic findings, C1QTNF6 was selected for experimental confirmation. C1QTNF6 was one of the key proteins found to be significantly down-regulated (adjusted *p*-value < 0.001) in both the Pre-Op and Post-Op groups when compared to the healthy controls. C1QTNF6 was selected for the ELISA validation based on its significant down-regulation observed in the proteomic analysis, its established role in extracellular matrix regulation and inflammation [16,17], and the availability of a commercial ELISA kit.

**Figure 9:**
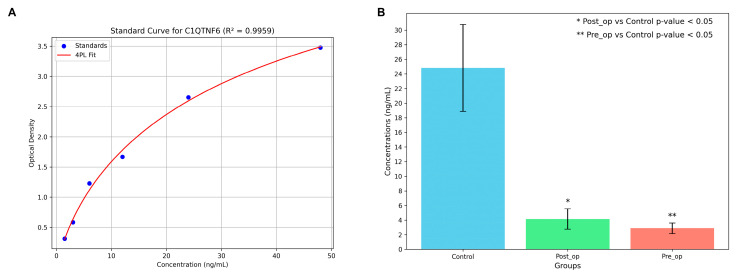
Experimental validation of C1QTNF6 expression by ELISA. (**A**) Standard curve for the quantification of C1QTNF6 using a four-parameter logistic (4PL) regression model. The curve was generated using serial dilutions of known standard concentrations, with corresponding OD values measured at 450 nm. The fitted curve demonstrated a high degree of accuracy with an R^2^ value of 0.9959. (**B**) Bar graph showing the measured C1QTNF6 concentrations (ng/mL) in control, Pre-Op, and Post-Op groups. Data is presented as mean ± SD, with individual data points representing independent patient samples. ELISA measurements were performed in duplicate. Our results showed the following concentrations (mean ± standard deviation): Control group: 24.82 ± 22.24; Pre-Op group: 2.88 ± 2.18; Post-Op group: 4.14 ± 4.45. Both patient groups showed significantly lower C1QTNF6 levels compared to healthy controls.

The concentrations of C1QTNF6 in the groups, along with the group-wise comparisons and the standard curve used for quantification, are presented in Figure 9. Independent samples *t*-tests between the control group and Pre-Op group, as well as between the control group and Post-Op group, both yielded *p*-values < 0.05. Consistent with our proteomic findings, the expression levels of C1QTNF6 were significantly lower in the OA patients compared to the healthy controls in both states.

## 3. Discussion

This study delineates systemic proteomic alterations associated with OA and their evolution six weeks after arthroplasty. By profiling the plasma before and after surgery against matched healthy controls, we identified (i) a stable OA-associated signature comprising 63 consistently up-regulated proteins and 23 down-regulated proteins across both time points and (ii) recovery-associated shifts unique to the post-operative phase. Together, this data suggests that surgery modulates aspects of systemic biology while leaving a core set of disease-linked perturbations largely intact.

Consistent with the well-established role of inflammation and ECM remodeling in OA pathogenesis, our data showed significant up-regulation of proteins such as TENT5D, WDR41, and HOXC9 in the Pre-Op patients. TENT5D belongs to the non-canonical poly(A) polymerase (TENT5/FAM46) family, whose members regulate post-transcriptional mRNA stability through cytoplasmic polyadenylation [18]. Genetic variants in FAM46A (TENT5A) have been previously associated with susceptibility to large-joint OA [19]. In contrast, FAM46C (TENT5C) has been identified as a frequently mutated gene in multiple myeloma and encodes an active non-canonical poly(A) polymerase with tumor-suppressive activity that regulates mRNA stability and cell proliferation [20]. These findings underscore functional diversity within the TENT5 family and indicate that individual members may exert distinct biological roles. However, the specific function of TENT5D remains poorly characterized. Experimental evidence demonstrates that TENT5D deficiency disrupts mRNA stability during spermatogenesis, supporting its role in post-transcriptional regulation [21]; nevertheless, to our knowledge, no direct association between TENT5D expression and OA or cartilage biology has been previously reported. Therefore, the marked elevation of circulating TENT5D observed in our cohort should be interpreted as a novel and exploratory proteomic finding rather than evidence of an established mechanistic link to OA.

WDR41 has been implicated in lysosomal homeostasis and regulation of innate immune signaling within the SMCR8–C9ORF72 complex in non-articular disease models [22]. Although its specific role in OA has not been investigated, dysregulated autophagy and innate immune activation are increasingly recognized contributors to OA pathogenesis [23,24]. Therefore, the elevated circulating WDR41 levels observed in our cohort may represent perturbations within these systemic regulatory axes; however, this interpretation remains hypothetical and requires validation in OA-specific experimental systems. HOXC9, a transcription factor involved in skeletal system morphogenesis, has also been linked to cartilage degeneration [25]. Together, the up-regulation of these proteins reflects the heightened cellular stress, tissue remodeling, and inflammatory activity characteristic of OA.

In addition to these up-regulated proteins, the Pre-Op group also exhibited alterations in molecules associated with apoptosis, signaling, and metabolic regulation, such as ANKRD26, ADCY8, and CTSD. ANKRD26 has been associated with platelet biology and inherited thrombocytopenia [26], which is consistent with the prominent enrichment of platelet activation and hemostasis pathways observed in our dataset (Supplementary Table S2). ADCY8, a calcium/calmodulin-sensitive adenylate cyclase, was significantly enriched in Relaxin signaling, platelet activation, and actin cytoskeleton-related pathways (Supplementary Tables S2 and S3). These pathway-level associations suggest altered second messenger signaling and platelet–immune interactions within the systemic context of OA. Notably, cAMP/CREB signaling has been shown to modulate chondrocyte autophagy and OA progression [27], supporting the potential relevance of altered cAMP-related pathways in OA pathophysiology. Similarly, elevated CTSD is consistent with its established role in ECM degradation and impaired autophagy, processes central to OA pathogenesis [28,29]. Conversely, down-regulation of structural proteins including COL6A1 and C1QTNF6 underscores a compromised ability to maintain cartilage stability and regulate immune responses [30,31]. Taken together, these Pre-Op alterations point toward a systemic environment dominated by catabolism, inflammation, and insufficient repair.

Following surgical intervention, our analysis revealed a partial normalization of several dysregulated proteins. For instance, AFM, CAPN11, GTF2E1, INTS6, ACTBL2, LDHB, and STAB1, which were significantly altered pre-operatively, approached levels comparable to healthy controls after surgery, suggesting that correction of biomechanical stress reduces systemic molecular disturbances and alleviates transcriptional strain, with partial restoration of nuclear homeostasis. These findings indicate that surgery not only restores joint function mechanically but also exerts systemic effects on molecular pathways related to cartilage homeostasis. However, proteins such as ORM2, B2M, and ATP6V0D1 remained dysregulated in the Post-Op group, highlighting that immune activation and metabolic stress persist during the recovery period and may contribute to variable patient outcomes [28,29,32].

Interestingly, we also identified proteins that were stable in Pre-Op patients but changed significantly in the Post-Op phase, such as CASP5, SAA1, FANCM, IGHG3, RAB34, SAMD4B, ACSL5, ARG1, DBH, DHRS11, FEN1, FGB, HBS1L, IGKV2-24, IGKV3-11, IQGAP2, PPP1R14A, R3HCC1, SERPINC1, and TAF1B. These likely represent acute responses to surgical trauma, including immune activation, apoptosis, and tissue remodeling. In particular, several of these newly altered proteins map to immune and coagulation programs: up-regulated SAA1, IGHG3, FANCM suggest enhanced acute-phase and humoral responses, whereas down-regulated FGB, ARG1, IGKV3-11, and IQGAP2 indicate a shift toward immune resolution and hemostatic rebalancing. The concomitant decrease in FGB and SERPINC1 further points to dynamic modulation within the fibrin-clot formation axis, potentially reflecting controlled attenuation of coagulation activity during recovery.

Fibrinogen (FGB) is a soluble plasma glycoprotein that serves as the precursor of fibrin and plays a pivotal role in platelet aggregation and the maintenance of plasma viscosity, thereby linking coagulation dynamics with inflammatory processes [33]. Consistent with synovial-compartment proteomic data, FGB in synovial fluid is reduced in OA relative to non-OA controls despite elevated circulating levels, and synovial FGB negatively correlates with pain and functional impairment. This compartmental divergence supports a model of local consumption, sequestration, or structural modification within the joint milieu [34]. In our data, the post-operative decline of FGB together with SERPINC1 aligns with this hemostatic rebalancing axis and may reflect recovery-phase adjustments in coagulation–inflammation crosstalk. Antithrombin III (SERPINC1) is a plasma serpin that inhibits thrombin and other activated serine proteases, with activity markedly potentiated by heparin/glycosaminoglycans [35]. In the joint compartment, antithrombin is detectable in synovial fluid, and elevated antithrombin–proteinase complexes have been reported in OA and RA, consistent with local regulation of coagulation pathways [36]. In our cohort, the Post-Op-specific decrease in SERPINC1 relative to controls may indicate perioperative consumption or altered GAG-mediated modulation during recovery, though mechanistic interpretations remain speculative pending targeted validation. Serum amyloid A1 (SAA1) is a major acute-phase protein that is highly expressed in response to inflammation and tissue injury. In OA, elevated SAA1 expression within the synovial environment contributes to a pro-inflammatory and catabolic state, facilitating matrix degradation and joint inflammation [37]. The post-operative emergence of SAA1 up-regulation in our dataset likely reflects a transient, repair-related immune response, consistent with short-term inflammasome activation and cytokine release rather than ongoing disease progression.

Taken together, this pattern is consistent with transient, recovery-phase proteomic remodeling superimposed on disease biology. From a translational standpoint, such post-operative emergent signals may function as an early-warning module for treatment response and secondary effects of surgery—serving as monitoring biomarkers to flag patients who are recovering as expected versus those who may require adjunct optimization (e.g., anti-inflammatory or antithrombotic strategies). These hypotheses merit prospective validation in larger cohorts and standardized follow-up protocols. Distinguishing such recovery-related responses from disease-specific alterations will be essential for future biomarker development, particularly when evaluating patient recovery trajectories and long-term outcomes.

Persistent proteomic alterations across both pre- and post-operative states delineate a core OA signature that remains refractory to surgical correction. The pathways enriched within this cluster converge on four major biological axes: platelet activation/coagulation, immune response, ECM remodeling, and metabolic–hormonal signaling. Persistent activation of platelet degranulation, hemostasis, and calcium-mediated secretion modules suggest a stable pro-thromboinflammatory tone that may contribute to endothelial stress and impaired resolution. Simultaneously, sustained enrichment of neutrophil degranulation and innate immune pathways implies ongoing low-grade inflammation and platelet–leukocyte crosstalk. The continuous up-regulation of ECM organization and collagen degradation components highlights incomplete structural recovery and supports the concept of a persistent remodeling phenotype in OA. Complementary involvement of IGF transport and hormonal signaling further indicates systemic recalibration of cellular signaling networks. Collectively, these enduring changes define a molecular footprint of OA that persists beyond mechanical correction, offering a candidate biomarker panel for disease monitoring and potential targets for adjunct anti-inflammatory or anti-thrombotic interventions.

In addition to the prominently up-regulated proteins, our data also revealed a distinct subset of proteins that were consistently down-regulated across both Pre-Op and Post-Op time points, suggesting a sustained suppression associated with the chronic OA phenotype. This group included proteins involved in diverse biological processes such as immune regulation–apoptosis (MST1), protease inhibition (SERPINA4, PZP), coagulation (F10), and cellular clearance pathways (GULP1), indicating that OA may be characterized not only by the activation of inflammatory and remodeling pathways but also by the attenuation of specific protective or homeostatic mechanisms.

MST1 has been implicated in chondrocyte apoptosis, inflammatory responses, and extracellular matrix degradation in OA. Notably, inhibition of MST1 has been shown to attenuate OA progression by promoting mitophagy and modulating the Nrf2–NF-κB signaling axis [38]. The balance between proteases and their inhibitors is increasingly being recognized as a critical component of OA pathogenesis. Members of the serpin family have been consistently reported as differentially regulated in omics-based analyses of OA tissues and synovial fluid, suggesting that their altered expression may contribute to a proteolytic microenvironment favoring cartilage degradation [35]. In a serum proteomic study, biomarkers indicative of knee OA were shown to discriminate patients from controls and were found to implicate complement and coagulation pathways, supporting the involvement of innate immunity in OA development [39]. In this context, alterations in coagulation-related proteins observed in our dataset, including F10, may reflect a broader dysregulation of the hemostatic–inflammatory balance in OA. GULP1 has been shown to play a regulatory role in chondrocyte growth arrest and differentiation through modulation of the TGF-β/SMAD2/3 signaling pathway. Experimental knockdown of GULP1 impairs SMAD2/3 activation, reduces p21 expression, and disrupts chondrogenic differentiation, highlighting its importance in maintaining chondrocyte homeostasis [40]. In this context, the persistent down-regulation of GULP1 observed in our dataset may reflect an impairment in cartilage homeostasis and regenerative capacity in OA.

Notably, transthyretin (TTR), a transport protein primarily involved in thyroid hormone and retinol binding, was consistently down-regulated in OA patients. Given that our pathway analysis (Supplementary Tables S2 and S3) linked TTR to the thyroid hormone synthesis pathway, this finding may reflect a broader dysregulation of endocrine–metabolic signaling in OA. Reduced TTR levels have also been associated with systemic inflammatory states and may represent a negative acute-phase response [41], suggesting that its suppression could be part of a chronic inflammatory milieu rather than a disease-specific isolated event. Beyond its canonical transport functions, accumulating evidence suggests that TTR may also participate in osteoarticular pathophysiology [42]. TTR deposits have been identified in the articular cartilage of OA patients and linked to inflammation and disease progression, while experimental studies further show that aggregated TTR can induce catabolic and inflammatory mediators in joint tissues, supporting a potential role in cartilage degeneration [43,44]. In parallel, alterations in circulating TTR forms have been reported in OA, including reduced levels of N-terminally truncated TTR in patients with active disease [45]. Interestingly, genetic deletion of TTR has been shown to exacerbate OA severity [44]. Taken together, these findings indicate that reduced circulating TTR levels in our cohort may not simply reflect a non-specific inflammatory response but could also be linked to complex alterations in cartilage metabolism, inflammatory signaling, and extracellular matrix homeostasis in OA.

The enrichment of the Relaxin signaling pathway at Pre-Op and Post-Op time points suggests that hormonal modulation of ECM dynamics may be related to OA. Recent evidence indicates that relaxin-2 can mitigate pro-inflammatory signaling and cellular senescence in human chondrocytes, potentially influencing critical degeneration and repair processes in OA tissues [46]. The presence of a significant thyroid hormone synthesis pathway supports the existence of an endocrine–metabolic component in OA. A recent Mendelian randomization and transcriptomic analysis has suggested a positive causal relationship between hyperthyroidism and OA. Triiodothyronine (T3) affects chondrocyte hypertrophy and ECM organization, processes closely linked to cartilage degeneration and joint remodeling [47]. This suggests that thyroid hormone signaling may contribute to cartilage catabolism and subchondral bone changes that characterize OA progression. Importantly, the term “Complement cascade regulation” is uniquely enriched in the Post-Op group, consistent with the idea that surgical intervention creates an immune regulatory signature. Current studies have shown that complement activation contributes to low-grade inflammatory processes in OA and that local production of C3 and other complement components is associated with pathological severity and synovial inflammation [48]. Furthermore, modulation of complement activity, for example, through inhibition of terminal complement complex formation, has been shown to reduce inflammation and matrix degradation in OA models, suggesting that this pathway plays a role in both disease pathogenesis and healing responses [49].

The enrichment of platelet activation and complement/coagulation cascades observed in our dataset is consistent with previously reported plasma proteomic signatures in OA. Complement and coagulation pathways have been identified as dominant components of a serum diagnostic peptide panel for knee OA [39,50], while Reactome enrichment characterized by platelet activation and neutrophil degranulation has been reported in targeted plasma proteomic analyses of OA [51]. Furthermore, coagulation-related pathways have been linked to disease outcomes, with extracellular vesicle-associated fibrinogen chains shown to predict radiographic OA progression [52]. The overlap of our findings with these independent studies supports the reproducibility of the systemic inflammatory and hemostatic signature in OA plasma. Rather than representing a novel mechanistic discovery, our results validate and extend this established model by confirming its persistence across both pre- and post-operative states. This temporal dimension suggests that modulation of complement and coagulation pathway activity reflects not only chronic disease processes but also dynamic post-operative immune adaptation.

Given the well-established molecular heterogeneity of OA, the interpretation of persistently elevated and post-surgery-specific proteins should be approached with caution. Emerging evidence supports the existence of distinct OA endotypes, including a hyperinflammatory phenotype characterized by complement activation and Fc receptor signaling, and a relatively hypoinflammatory structural phenotype dominated by extracellular matrix remodeling [53,54]. Recent plasma proteomic studies further demonstrate that pre-operative molecular profiles can stratify OA patients into biologically distinct response subgroups. Pre-operative inflammatory clustering has been associated with divergent pain trajectories following arthroplasty [55], while inflammatory biomarker profiling has also been reported to correlate with differential post-operative outcomes in OA [56]. These findings indicate that baseline immune and molecular states may significantly influence both post-operative proteomic patterns and clinical outcomes. In this context, given the small cohort size and the inclusion of both knee and hip arthroplasty cases, the observed distinction between persistent and surgery-responsive proteins may, at least in part, reflect underlying endotype heterogeneity rather than a purely surgery-driven biological transition. To disentangle baseline disease heterogeneity from true surgery-induced proteomic reorganization, future studies incorporating systematic pre-operative immunophenotyping and larger, anatomically stratified cohorts will be essential.

The detection of several proteins typically considered intracellular at elevated levels in depleted plasma warrants further biological interpretation. Notably, our Gene Ontology enrichment analysis (Supplementary Tables S2 and S3) identified “extracellular exosome” and “blood microparticle” as significantly enriched cellular components. This observation is supported by a previous study demonstrating that extracellular vesicles (EVs)—including exosomes, microvesicles, and apoptotic bodies—can encapsulate and transport intracellular proteins and other bioactive cargo into the extracellular space and circulation [57]. Furthermore, circulating microparticles are known to originate from activated, stressed, or apoptotic cells through membrane budding processes, providing a well-established cellular mechanism for the release of intracellular components into the bloodstream [58]. These findings provide a plausible mechanistic explanation, suggesting that such proteins may enter the circulation via extracellular vesicle release, including exosome shedding, microparticle formation, or apoptotic leakage from stressed or damaged joint-associated cells. This joint-derived vesicle hypothesis is consistent with accumulating evidence that extracellular vesicles are key mediators of intercellular communication within the joint, facilitating crosstalk among chondrocytes, synovial fibroblasts, and mesenchymal stem cells; importantly, in OA, EV cargo (including proteins) undergoes pathological changes linked to inflammation and matrix degradation, supporting their biological relevance as disease-related carriers and potential biomarkers [59]. In line with this concept, recent plasma EV proteomics in knee OA demonstrated a substantial overlap between synovial fluid and plasma EV peptides, with correlated EV-associated signals enriched for immune, inflammatory, and complement-related processes, indicating that circulating EV profiles can reflect joint-associated pathophysiology and system-level immune signatures relevant to OA progression [52].

At the same time, we acknowledge that an alternative explanation may involve technical factors, particularly non-specific enrichment effects introduced during the high-abundance protein depletion process. Such methodological artifacts could potentially contribute to the detection of proteins not typically abundant in plasma. Indeed, previous studies have highlighted that commonly used depletion strategies, including immunoaffinity-based and dye–ligand approaches, are susceptible to non-specific binding and unintended co-depletion or enrichment of non-target proteins, thereby potentially distorting the representation of low-abundance proteomic profiles [60]. Given these considerations, further orthogonal validation using targeted approaches, such as ELISA or targeted mass spectrometry, is warranted for selected proteins to confirm their biological relevance and potential clinical utility. In our study, the ELISA results confirmed significantly lower C1QTNF6 levels in OA patients compared to healthy controls, supporting the robustness of the proteomic findings. This validation provides additional confidence in the biological relevance of at least a subset of the identified protein signatures.

Cartilage acidic protein 1 (CRTAC1), one of the most consistently replicated plasma biomarkers in OA [61,62,63], and cartilage oligomeric matrix protein (COMP), a well-established marker of cartilage turnover and joint tissue remodeling [64,65,66], were both detected in our dataset; however, neither met the predefined criteria for differential expression. Specifically, CRTAC1 did not reach statistical significance in either comparison (Pre-Op vs. control: *p* = 0.63, log_2_FC = 0.13; Post-Op vs. control: *p* = 0.92, log_2_FC = 0.04), while COMP, although showing statistical significance in the Post-Op comparison (*p* = 0.01), did not meet the fold-change threshold at either time point (Pre-Op: log_2_FC = −0.30; Post-Op: log_2_FC = −0.64). This apparent discrepancy with prior literature may reflect differences in cohort size, disease stage, or sample characteristics, as well as potential technical factors such as depletion strategies that may reduce the detectability of low-abundance cartilage-derived proteins. These findings highlight the heterogeneity of OA biomarker expression and underscore the need for cautious interpretation when extrapolating proteomic signatures across different study designs. Importantly, these observations do not contradict the established relevance of CRTAC1 and COMP in OA, but rather emphasize the context-dependent nature of biomarker expression.

This study has several limitations that should be considered when interpreting the findings. First, the relatively small sample size (*n* = 18; 8 OA patients and 10 controls) limits the statistical power of the analysis and increases the risk of false-positive findings, despite the application of multiple testing correction. Accordingly, the identified protein signatures should be interpreted as preliminary and exploratory rather than definitive biomarkers. Second, the study design includes only a single post-operative time point at six weeks, which primarily reflects early recovery and acute-phase responses rather than long-term molecular remodeling following arthroplasty. Longitudinal studies with extended follow-up are required to better characterize the temporal dynamics of proteomic changes in OA. Third, the cohort comprises both knee and hip arthroplasty patients without subgroup-specific analyses, potentially masking joint-specific molecular signatures and contributing to biological heterogeneity. Future studies with larger cohorts should incorporate stratified analyses to address potential site-specific differences. Fourth, although high-abundance protein depletion enhances the detection of lower-abundance proteins, it may also introduce technical biases, including non-specific protein loss or enrichment, which could influence the observed proteomic profiles. Fifth, while selected findings were supported by targeted ELISA validation for C1QTNF6, the majority of identified proteins have not yet undergone orthogonal validation. Therefore, further confirmation using targeted mass spectrometry or immunoassay-based approaches is warranted. Taken together, these limitations emphasize that the present findings should be viewed as hypothesis-generating and require validation in larger, independent, and longitudinal cohorts.

## 4. Materials and Methods

### 4.1. Study Design and Participant Selection

This study was designed to investigate the proteomic alterations in OA patients by comparing Pre-Op and Post-Op blood samples to those from healthy controls. A total of 18 participants were included in the study, comprising 8 OA patients scheduled for surgical intervention (either total knee arthroplasty for gonarthrosis or total hip arthroplasty for coxarthrosis) and 10 healthy control subjects. All biochemical measurements were performed as part of routine clinical assessment using automated analyzers in the central hospital laboratory. Data was retrieved from the laboratory information system.

#### 4.1.1. Inclusion Criteria

OA patients were selected based on clinical diagnosis criteria, including radiographic evidence of advanced OA requiring surgical intervention. The patient group included individuals diagnosed with gonarthrosis or coxarthrosis, with some patients undergoing total knee arthroplasty and others undergoing total hip arthroplasty. The patient cohort consisted of 4 women and 4 men, aged between 18 and 65 years. Healthy controls were matched to OA patients based on age, sex, and body mass index, with no history of OA or other inflammatory diseases. All participants provided written informed consent prior to inclusion in the study.

#### 4.1.2. Ethical Approval

The study protocol was approved by the Hamidiye Scientific Research Ethics Committee of the University of Health Sciences, Türkiye, under the registration number 23/217. The study was conducted in accordance with the Helsinki Declaration and Good Clinical Practice guidelines. All participants provided written informed consent before enrollment in the study.

### 4.2. Sample Collection and Preparation

#### 4.2.1. Blood Sample Collection

Blood samples were collected from OA patients at two time points: pre-operatively (within one week before surgery) and post-operatively (six weeks after surgery). Blood samples from healthy controls were collected at a single time point. Approximately 5 mL of blood was drawn from each participant via venipuncture and collected in BD Vacutainer tubes containing K2 EDTA. Each tube contained approximately 9 mg of EDTA, which was sufficient to prevent blood clotting and ensured the stability of the blood samples for subsequent analysis.

#### 4.2.2. Sample Processing

Blood samples were immediately centrifuged at 2000× *g* for 15 min at 4 °C to separate plasma. Plasma samples were aliquoted and stored at −80 °C until proteomic analysis.

### 4.3. Proteomic Analysis

#### 4.3.1. Protein Extraction and Quantification

Albumin, IgA, IgD, IgE, IgG (including light chains), IgM, Alpha-1-acid glycoprotein, Alpha-1-antitrypsin, Alpha-2-macroglobulin, Apolipoprotein A1, Fibrinogen, Haptoglobin, and Transferrin proteins, which were present at high levels in serum samples, were removed using the High-Select™ Top14 Abundant Protein Depletion Resin kit (A36370, Thermo Fisher Scientific, Waltham, MA, USA). High-abundance proteins were removed using a depletion spin column according to the manufacturer’s protocol. The sample was added to the resin slurry, thoroughly mixed, and incubated with gentle end-over-end agitation for 10 min at room temperature to ensure binding of target proteins. After centrifugation at 1000× *g* for 2 min, the filtrate containing depleted proteins in 10 mM PBS and 0.02% sodium azide (pH 7.4) was collected for further processing. The protein concentration was measured using the Qubit Protein Assay Kit (Q33212, Invitrogen, Life Technologies, Waltham, MA, USA), which is specifically designed for use with the Qubit 3.0 fluorometer (Q33216, Invitrogen, Life Technologies, Waltham, MA, USA). The fluorometer was calibrated using the standards provided in the assay kit according to the manufacturer’s instructions. This method ensures accurate and reproducible quantification of protein concentrations by employing a fluorescence-based approach that is highly sensitive to the protein content in the samples.

#### 4.3.2. Protein Digestion and Labeling

Samples were homogenized in 50 mM ammonium bicarbonate buffer (pH 7.8) (S2454-200ML, Sigma Aldrich, St. Louis, MO, USA) containing a protease inhibitor cocktail (ab270061, Expedeon, Heidelberg, Germany), and lysed at 95 °C in a protein extraction reagent kit (UPX Universal; Expedeon, Heidelberg, Germany). The samples were incubated for one hour at 4 °C after the lysis step. According to the manufacturer’s protocol, protein concentrations were determined using a Qubit 3.0 Fluorometer (Q33216, Invitrogen, Life Technologies, Waltham, MA, USA). Following homogenization, the FASP (Filter-Aided Sample Preparation) Protein Digestion Kit (ab270519, Abcam, Cambridge, UK) was used to generate tryptic peptides according to the manufacturer’s instructions. Protein solution, containing 50 μg of protein in 30 μL, were filtered using 6 M urea in a 30 kDa cutoff spin column. After this step, samples were alkylated with 10 mM iodoacetamide in the dark for 20 min at room temperature. Then, samples were incubated overnight with MS-grade trypsin protease (ratio 1:100, 90,057, Thermo Scientific, Waltham, MA, USA) at 37 °C. The following day, peptides were eluted from the columns and lyophilized. After the lyophilization process, the peptides were suspended in 0.1% formic acid (1,002,642,510, Merck, Darmstadt, Germany) and diluted to 100 ng/μL before injecting into the LC–MS/MS system (ACQUITY UPLC M-Class coupled to a SYNAPT G2-Si high-definition mass spectrometer (Waters, Milford, MA, USA)).

#### 4.3.3. Mass Spectrometry Analysis

LC–MS/MS and protein identification were performed with minor modifications according to previously published protocols [67]. Briefly, samples were loaded onto the ACQUITY UPLC M-Class coupled to a SYNAPT G2-Si high-definition mass spectrometer (Waters, Milford, MA, USA). To equilibrate the columns, 97% of the mobile phase (0.1% formic acid in LC-MS grade water) was used, and the column temperature was set to 55 °C. Ninety-minute gradient elution from the ACQUITY UPLC M-Class Symmetry C18 trap column (180 µm × 20 mm; 186007496, Waters) to the analytic column (ACQUITY UPLC M-Class HSS T3 Column, 100Å, 1.8 µm, 75 µm × 250 mm; 186007474, Waters) at 0.3 µL/min flow rate with a gradient from 4% to 40% hypergrade acetonitrile (100029, Merck) containing 0.1% formic acid (*v*/*v*) was used for the peptide separation [68]. Positive ion modes of MS and MS/MS scans with 0.6 s cycle time were performed sequentially. Ten volts were set as low collision energy (CE) and 30 V as high CE. Ion mobility separation (IMS) was used for the ion separation. A wave velocity was ramped from 1000 m/s to 55 m/s over the entire IMS cycle. The release time for mobility trapping was 500 μs, and trap height was set to 15 V. IMS wave delay was 1000 μs for the mobility separation after trap release. All the ions within the 50–1900 *m*/*z* range were fragmented in resolution mode without any precursor ion preselection. A total of 100 fmol/μL Glu-1-fibrinopeptide B (186007091-2, Waters, Milford, MA, USA) was used for lock mass reference with a 60 s interval to observe the mass stability.

### 4.4. Enzyme-Linked Immunosorbent Assay (ELISA)

Human C1QTNF6 levels were quantified using a commercially available ELISA kit (Human C1QTNF6, Catalog No: E5090Hu, Bioassay Technology Laboratory, BT Lab, Shanghai, China). The assay is based on the double-antibody sandwich ELISA principle. Microplate wells were pre-coated with an antibody specific to human C1QTNF6. Standards and samples were pipetted into the wells, allowing C1QTNF6 in the samples to bind to the immobilized antibody. Subsequently, a biotin-labeled detection antibody specific to C1QTNF6 was added, followed by the addition of streptavidin conjugated to horseradish peroxidase (HRP), forming an antibody–antigen–antibody “sandwich” complex. After incubation, unbound components were removed by washing. Next, a substrate solution was added, which reacted with the HRP enzyme to produce a colorimetric change. The enzymatic reaction was terminated by the addition of a stop solution, and optical density (OD) was measured at 450 nm using a BioTek Synergy HTX Multi-Mode Microplate Reader (BioTek Instruments, Winooski, VT, USA). All washing steps were performed using a BioTek 405 LS Microplate Washer (BioTek Instruments, Winooski, VT, USA). All reagents, standards, and samples were prepared and handled according to the manufacturer’s protocol.

The OD values of both standards and samples were corrected by subtracting the blank (zero standard) absorbance. A four-parameter logistic (4PL) curve was generated using a log–log plot, with standard concentrations on the x-axis and corresponding OD values on the y-axis. The concentrations of C1QTNF6 in the samples were calculated by interpolating their OD values against the standard curve.

### 4.5. Bioinformatics and Statistical Analysis

Data was analyzed using Progenesis-QI for proteomics software (v2, Milford, MA, Waters) to identify and quantify peptides. All detected proteins were evaluated with at least 2 unique peptide sequences, and the expression ratio was calculated. Kyoto Encyclopedia of Genes and Genomes (KEGG) and Reactome pathway analyses, along with Gene Ontology (GO) enrichment analysis, were conducted using the Database for Annotation, Visualization, and Integrated Discover (DAVID; https://davidbioinformatics.nih.gov/tools.jsp, v2023q4, accessed on 17 March 2026). After pathway analysis, enrichment bubble plots were created for the top pathways with enrichment scores, *p*-values, and count values using the online SRplot program (available at https://www.bioinformatics.com.cn/), accessed on 17 March 2026 [69]. Protein–protein interaction (PPI) analysis was performed using the STRING database (https://string-db.org/, accessed on 17 March 2026, Search Tool for the Retrieval of Interacting Genes/Proteins, Swiss Institute of Bioinformatics, Switzerland). The confidence level was set at 0.7. To evaluate the protein lists obtained as a result of proteomics analysis, Babelomics 5 software was used for FDR (false discovery rate)-corrected paired *t*-test, and limma analysis for overall group comparison was performed using Bioconductor software (Version 3.20, R 4.4.2) [70,71,72]. For cross-sectional contrasts between patient groups and controls, independent samples *t*-tests were applied. Multiple testing correction was performed using the Benjamini–Hochberg method to control the FDR and ensure robust identification of significant proteins. In all analyses, differential abundance was defined using a dual threshold of FDR-adjusted *p* < 0.05 and |log_2_ (fold change)| > 1, applied uniformly across all comparisons.

## 5. Conclusions

Overall, this exploratory study provides a plasma proteomic overview of OA by integrating pre- and post-operative profiling, identifying both persistent and surgery-associated molecular alterations. The enrichment of platelet activation, coagulation, immune, and extracellular matrix-related pathways suggests that OA may be associated with a sustained systemic thromboinflammatory and remodeling-related signature detectable in circulation. In parallel, the emergence of acute-phase and complement-related changes following surgery likely reflects early post-operative immune adaptation superimposed on the chronic disease background. These findings support the concept that OA involves interconnected inflammatory, hemostatic, and tissue remodeling processes that extend beyond the local joint environment, underscoring the potential of circulating proteomic biomarkers to reflect both disease activity and post-operative recovery trajectories. However, given the limited cohort size and single post-operative time point, the identified proteomic signatures should be interpreted as preliminary and hypothesis-generating.

Future studies in larger, longitudinal, and stratified cohorts will be required to validate these observations and to clarify the temporal dynamics of systemic proteomic changes before and after arthroplasty. Integration of plasma with synovial fluid analyses and targeted validation approaches may help delineate systemic versus local mechanisms. Further mechanistic investigation of selected candidate proteins, including TENT5D, WDR41, HOXC9, ANKRD26, ADCY8, CTSD, COL6A1, C1QTNF6, FGB, SERPINC1, and SAA1, which are broadly involved in development, signaling, and extracellular matrix biology, could refine our understanding of coagulation–inflammation interplay in OA biology. Incorporating multi-omics layers may further refine the molecular map of OA and its modulation after surgery. With appropriate validation, such efforts may contribute to the future development of more precise molecular frameworks for monitoring disease activity, informing personalized therapeutic strategies, and tracking post-operative recovery.

## Figures and Tables

**Table 1:** Biochemical parameters measured in the control, Pre-Op, and Post-Op OA patients. The table includes mean values and standard deviations (mean ± SD) for each group; *p*-values were calculated using independent samples *t*-test. Reference ranges are provided for comparison.

Parameter	Control Group	Pre-Op Group	*p*-Value (Control vs. Pre-Op)	Post-Op Group	Reference Range
WBC (×10^9^/L)	6.26 ± 1.28	8.05 ± 1.31	0.01	10.14 ± 1.71	4.0–11.0
RBC (×10^12^/L)	4.76 ± 0.27	4.61 ± 0.41	>0.05	3.64 ± 0.38	4.2–6.1
HGB (g/dL)	13.84 ± 1.18	12.63 ± 1.35	>0.05	10.05 ± 1.22	12.0–17.5
HCT (%)	41.66 ± 3.43	40.33 ± 4.88	>0.05	31.58 ± 3.34	36–53
PLT (×10^9^/L)	241.13 ± 73.09	235.38 ± 58.54	>0.05	229.38 ± 38.81	150–450
MCV (fL)	87.55 ± 4.83	86.85 ± 7.20	>0.05	86.96 ± 7.66	80–100
MPV (fL)	8.48 ± 0.5	9.51 ± 1.16	0.046	9.55 ± 0.90	7.5–11.5
Neutrophils (×10^9^/L)	4.54 ± 1.5	4.58 ± 0.96	>0.05	7.44 ± 2.08	2.0–7.0
Lymphocytes (×10^9^/L)	2.52 ± 0.5	2.47 ± 0.75	>0.05	1.82 ± 0.71	1.0–3.5
Monocytes (×10^9^/L)	0.51 ± 0.1	0.48 ± 0.10	>0.05	0.71 ± 0.22	0.2–1.0
Glucose (mg/dL)	89.97 ± 5.2	111.58 ± 31.15	>0.05	150.23 ± 44.71	70–100
Urea (mg/dL)	25.13 ± 4.3	38.79 ± 23.40	>0.05	23.55 ± 9.83	10–50
Creatinine (mg/dL)	0.88 ± 0.11	0.88 ± 0.36	>0.05	0.72 ± 0.16	0.7–1.2 (M), 0.5–0.9 (F)
ALT (U/L)	20.04 ± 5.1	16.64 ± 8.76	>0.05	15.75 ± 8.13	7–56
AST (U/L)	19.98 ± 4.3	20.50 ± 9.05	>0.05	18.60 ± 2.90	10–40
GGT (U/L)	12.24 ± 6.12	21.75 ± 11.97	>0.05	21.25 ± 3.94	0–60 (M), 0–36 (F)
Cholesterol (mg/dL)	167.92 ± 39.49	208.38 ± 28.28	0.022	196.38 ± 27.74	90–200
HDL (mg/dL)	53.33 ± 11.48	52.76 ± 12.97	>0.05	43.53 ± 10.01	40–60
LDL (mg/dL)	94.23 ± 31.37	115.44 ± 16.88	>0.05	124.71 ± 17.02	<100
Triglycerides (mg/dL)	103.31 ± 90.85	197.38 ± 69.52	0.025	146.75 ± 44.96	<150
Total Protein (g/L)	70.03 ± 2.8	74.20 ± 4.72	0.048	74.00 ± 4.53	60–80
Albumin (g/L)	42.02 ± 3.42	43.91 ± 3.74	>0.05	44.25 ± 3.89	35–50
Calcium (mg/dL)	9.48 ± 0.27	9.59 ± 0.43	>0.05	9.69 ± 0.16	8.5–10.5
Sodium (mmol/L)	139.8 ± 2.29	138.38 ± 2.20	>0.05	138.00 ± 1.41	135–145
Potassium (mmol/L)	4.22 ± 0.27	4.36 ± 0.53	>0.05	3.93 ± 0.11	3.5–5.0
Phosphorus (mg/dL)	3.51 ± 0.29	3.36 ± 0.61	>0.05	3.05 ± 0.64	2.5–4.5
Ferritin (ng/mL)	92.65 ± 61.96	64.10 ± 38.69	>0.05	70.87 ± 55.54	20–500 (M), 20–200 (F)
Procalcitonin (ng/mL)	0.11 ± 0.07	0.05 ± 0.03	>0.05	0.06 ± 0.04	<0.5
CRP (mg/L)	1.03 ± 0.37	8.25 ± 6.91	0.02	3.82 ± 3.23	<5

**Table 2:** Differentially expressed proteins showing ≥5-fold change relative to healthy controls in Pre- and Post-operative OA patients. Fold-change values and *p*-values are shown for Pre-Op vs. control and Post-Op vs. control comparisons. Positive fold-change indicates up-regulation, and values below 1 indicate down-regulation relative to controls.

Protein	Fold-Change Values		*p* Values	
	Pre-Op vs. Control	Post-Op vs. Control	Pre-Op vs. Control	Post-Op vs. Control
HEMK1	12.20	11.39	<0.001	<0.001
AGAP6	0.10	0.17	<0.001	0.001
CXCL4V1	5.45	6.43	<0.001	<0.001
CHTF18	7.25	7.59	<0.001	<0.001
ATP6V0D1	6.18	6.09	<0.001	<0.001
KLK15	0.08	0.05	<0.001	<0.001
CTSD	17.38	14.92	<0.001	<0.001
ACTB	5.26	5.44	<0.001	<0.001
B2M	6.47	7.20	<0.001	<0.001
YWHAZ	0.01	0.19	<0.001	0.014
HERC3	0.10	0.20	0.001	0.001
EPHA10	0.07	0.04	0.001	<0.001
ZNF546 *	13.57	4.92	0.003	<0.001
TTLL11 *	0.21	0.20	0.001	0.001
USP13 *	5.60	3.70	0.001	0.007
ANKRD26	11.54	10.30	<0.001	<0.001
TAGLN2 *	0.08	0.24	<0.001	0.026
ORM2	6.46	8.59	<0.001	<0.001
C1QTNF6	0.14	0.12	<0.001	<0.001
PDHB	6.64	5.83	<0.001	<0.001
TENT5D	50.14	41.05	<0.001	<0.001
WDR41	20.92	23.50	<0.001	<0.001
PROC	6.77	5.80	<0.001	<0.001
SPEF2	5.83	5.23	<0.001	<0.001
CCDC150 *	5.82	4.93	<0.001	<0.001
HOXC9	24.75	17.38	<0.001	<0.001
TRIM52	0.09	0.10	<0.001	<0.001
GP5	11.63	12.93	<0.001	<0.001
PPP3R2	18.13	7.59	<0.001	0.001
PBXIP1	13.36	12.76	0.001	0.001
CHPF *	4.34	5.05	0.002	0.001
ADCY8	11.61	10.48	0.009	<0.001
COL6A1 *	0.20	0.16	<0.001	<0.001
HBS1L **	0.09	0.05	0.06	0.013

Proteins marked with a single asterisk (*) exceed the ≥5-fold change threshold in only one comparison (either Pre-Op vs. control or Post-Op vs. control). Non-marked proteins satisfy both the fold-change (≥5-fold) and statistical significance (*p* < 0.05) criteria at both time points. Proteins marked with a double asterisk (**) exceed the ≥5-fold threshold at both time points but reach statistical significance (*p* < 0.05) in only one comparison.

## Data Availability

The mass spectrometry data generated in this study have been deposited in the Japan Proteome Standard (jPOST) Repository/Database, which is one of the ProteomeXchange consortium with the dataset ID PXD075088.

## Supplementary Materials

The following supporting information can be downloaded at: https://www.mdpi.com/article/10.3390/ijms27062862/s1.

## Abbreviations

The following abbreviations are used in this manuscript:

OA	Osteoarthritis
Pre-Op	Pre-Operative
Post-Op	Post-Operative
FASP	Filter-Aided Sample Preparation
CE	Collision Energy
IMS	Ion Mobility Separation
ELISA	Enzyme-Linked Immunosorbent Assay
HRP	Horseradish Peroxidase
OD	Optical Density
4PL	Four-Parameter Logistic
KEGG	Kyoto Encyclopedia of Genes and Genomes
GO	Gene Ontology
DAVID	Database for Annotation, Visualization, and Integrated Discovery
PPI	Protein–Protein Interaction
STRING	Search Tool for the Retrieval of Interacting Genes/Proteins
FDR	False Discovery Rate
FC	Fold Change
FGB	Fibrinogen
SERPINC1	Antithrombin III
SAA1	Serum Amyloid A1
TTR	Transthyretin
EV	Extracellular Vesicle
CRTAC1	Cartilage Acidic Protein 1
COMP	Cartilage Oligomeric Matrix Protein

## References

[1] World Health Organization (2023). Osteoarthritis. https://www.who.int/news-room/fact-sheets/detail/osteoarthritis.

[2] GBD 2021 Osteoarthritis Collaborators (2023). Global, regional, and national burden of osteoarthritis, 1990–2020 and projections to 2050: A systematic analysis for the Global Burden of Disease Study 2021. Lancet Rheumatol..

[3] Wong A.Y., Samartzis D., Maher C. (2023). The global burden of osteoarthritis: Past and future perspectives. Lancet Rheumatol..

[4] Kiełbowski K., Herian M., Bakinowska E., Banach B., Sroczyński T., Pawlik A. (2023). The Role of Genetics and Epigenetic Regulation in the Pathogenesis of Osteoarthritis. Int. J. Mol. Sci..

[5] Perrot S., Anne-Priscille T. (2023). Pain in osteoarthritis from a symptom to a disease. Best Pract. Res. Clin. Rheumatol..

[6] Sheng W., Wang Q., Qin H., Cao S., Wei Y., Weng J., Yu F., Zeng H. (2023). Osteoarthritis: Role of Peroxisome Proliferator-Activated Receptors. Int. J. Mol. Sci..

[7] Tang S., Zhang C., Oo W.M., Fu K., Risberg M.A., Bierma-Zeinstra S.M., Neogi T., Atukorala I., Malfait A.M., Ding C. (2025). Osteoarthritis. Nat. Rev. Dis. Primers.

[8] Mobasheri A., Thudium C.S., Bay-Jensen A.C., Maleitzke T., Geissler S., Duda G.N., Winkler T. (2023). Biomarkers for osteoarthritis: Current status and future prospects. Best Pract. Res. Clin. Rheumatol..

[9] Hunter D.J., Nevitt M., Losina E., Kraus V. (2014). Biomarkers for osteoarthritis: Current position and steps towards further validation. Best Pract. Res. Clin. Rheumatol..

[10] Coleman L.J., Byrne J.L., Edwards S., O’Hara R. (2025). Advancing Early Detection of Osteoarthritis Through Biomarker Profiling and Predictive Modelling: A Review. Biologics.

[11] Welhaven H.D., Welfley A.H., June R.K. (2025). Osteoarthritis Year in Review 2024: Molecular biomarkers of osteoarthritis. Osteoarthr. Cartilage.

[12] Li G., Stampas A., Komatsu Y., Gao X., Huard J., Pan S. (2024). Proteomics in orthopedic research: Recent studies and their translational implications. J. Orthop. Res..

[13] Vrazas V., Katsani K.R. (2024). Proteomics. Handbook of Molecular Biotechnology.

[14] Ridker P.M. (2022). Proteomics for the prediction and prevention of atherosclerotic disease. Eur. Heart J..

[15] Zubair M., Wang J., Yu Y., Faisal M., Qi M., Shah A.U., Feng Z., Shao G., Wang Y., Xiong Q. (2022). Proteomics approaches: A review regarding an importance of proteome analyses in understanding the pathogens and diseases. Front. Vet. Sci..

[16] Xu C., Sarver D.C., Lei X., Sahagun A., Zhong J., Na C.H., Rudich A., Wong G.W. (2024). CTRP6 promotes the macrophage inflammatory response, and its deficiency attenuates LPS-induced inflammation. J. Biol. Chem..

[17] Xu E., Yin C., Yi X., Liu Y. (2020). Knockdown of CTRP6 inhibits high glucose-induced oxidative stress, inflammation and extracellular matrix accumulation in mesangial cells through regulating the Akt/NF-κB pathway. Clin. Exp. Pharmacol. Physiol..

[18] Kuchta K., Muszewska A., Knizewski L., Steczkiewicz K., Wyrwicz L.S., Pawlowski K., Rychlewski L., Ginalski K. (2016). FAM46 proteins are novel eukaryotic non-canonical poly(A) polymerases. Nucleic Acids Res..

[19] Etokebe G.E., Jotanovic Z., Mihelic R., Mulac-Jericevic B., Nikolic T., Balen S., Sestan B., Dembic Z. (2015). Susceptibility to large-joint osteoarthritis (hip and knee) is associated with BAG6 rs3117582 SNP and the VNTR polymorphism in the second exon of the FAM46A gene on chromosome 6. J. Orthop. Res..

[20] Mroczek S., Chlebowska J., Kuliński T.M., Gewartowska O., Gruchota J., Cysewski D., Liudkovska V., Borsuk E., Nowis D., Dziembowski A. (2017). The non-canonical poly(A) polymerase FAM46C acts as an onco-suppressor in multiple myeloma. Nat. Commun..

[21] Cong J., Yang Y., Wang X., Shen Y., Qi H.T., Liu C., Tang S., Wu S., Tian S., Zhou Y. (2022). Deficiency of X-linked TENT5D causes male infertility by disrupting the mRNA stability during spermatogenesis. Cell Discov..

[22] McAlpine W., Sun L., Wang K.W., Liu A., Jain R., San Miguel M., Wang J., Zhang Z., Hayse B., McAlpine S.G. (2018). Excessive endosomal TLR signaling causes inflammatory disease in mice with defective SMCR8-WDR41-C9ORF72 complex function. Proc. Natl. Acad. Sci. USA.

[23] Caramés B., Taniguchi N., Otsuki S., Blanco F.J., Lotz M. (2010). Autophagy is a protective mechanism in normal cartilage, and its aging-related loss is linked with cell death and osteoarthritis. Arthritis Rheumatol..

[24] Scanzello C.R., Goldring S.R. (2012). The role of synovitis in osteoarthritis pathogenesis. Bone.

[25] Aref-Eshghi E., Zhang Y., Liu M., Harper P.E., Martin G., Furey A., Green R., Sun G., Rahman P., Zhai G. (2015). Genome-wide DNA methylation study of hip and knee cartilage reveals embryonic organ and skeletal system morphogenesis as major pathways involved in osteoarthritis. BMC Musculoskelet. Disord..

[26] Pippucci T., Savoia A., Perrotta S., Pujol-Moix N., Noris P., Castegnaro G., Pecci A., Gnan C., Punzo F., Marconi C. (2011). Mutations in the 5′ UTR of ANKRD26, the ankirin repeat domain 26 gene, cause an autosomal-dominant form of inherited thrombocytopenia, THC2. Am. J. Hum. Genet..

[27] Zhang H., Li X., Li Y., Yang X., Liao R., Wang H., Yang J. (2022). CREB Ameliorates Osteoarthritis Progression Through Regulating Chondrocytes Autophagy via the miR-373/METTL3/TFEB Axis. Front. Cell Dev. Biol..

[28] Shi H., Li B., Zhang D., Han H., He H., Zhu J., Wang H., Chen L. (2023). Autophagy inhibition mediated by intrauterine miR-1912-3p/CTSD programming participated in the susceptibility to osteoarthritis induced by prenatal dexamethasone exposure in male adult offspring rats. FASEB J..

[29] Piñeiro-Ramil M., Gómez-Seoane I., Rodríguez-Cendal A.I., Sanjurjo-Rodríguez C., Riva-Mendoza S., Fuentes-Boquete I., De Toro-Santos J., Señarís-Rodríguez J., Díaz-Prado S. (2025). Disease-Associated Signatures Persist in Extracellular Vesicles from Reprogrammed Cells of Osteoarthritis Patients. Int. J. Mol. Sci..

[30] Alexopoulos L.G., Youn I., Bonaldo P., Guilak F. (2009). Developmental and osteoarthritic changes in Col6a1-knockout mice: Biomechanics of type VI collagen in the cartilage pericellular matrix. Arthritis Rheumatol..

[31] Hu B., Qian X., Qian P., Xu G., Jin X., Chen D., Xu L., Tang J., Wu W., Li W. (2022). Advances in the functions of CTRP6 in the development and progression of the malignancy. Front. Genet..

[32] Zhang X., Wang C., Zhao J., Xu J., Geng Y., Dai L., Huang Y., Fu S.C., Dai K., Zhang X. (2017). miR-146a facilitates osteoarthritis by regulating cartilage homeostasis via targeting Camk2d and Ppp3r2. Cell Death Dis..

[33] Beydoun H.A., Archer D.F., Zonderman A.B., Beydoun M.A. (2015). Interrelationships of Sex, Physician-Diagnosed Arthritis, Chronic Inflammation, and Physical Functioning in the Third National Health and Nutrition Examination Surveys. Gerontol. Geriatr. Med..

[34] Rocchetti M.T., Bizzoca D., Moretti L., Ragni E., Moretti F.L., Vicenti G., Solarino G., Rizzello A., Petruzzella V., Palese L.L. (2023). A Gel-Based Proteomic Analysis Reveals Synovial α-Enolase and Fibrinogen β-Chain Dysregulation in Knee Osteoarthritis: A Controlled Trial. J. Pers. Med..

[35] Wilkinson D.J. (2021). Serpins in cartilage and osteoarthritis: What do we know?. Biochem. Soc. Trans..

[36] Furmaniak-Kazmierczak E., Cooke T.D., Manuel R., Scudamore A., Hoogendorn H., Giles A.R., Nesheim M. (1994). Studies of thrombin-induced proteoglycan release in the degradation of human and bovine cartilage. J. Clin. Investig..

[37] de Seny D., Cobraiville G., Charlier E., Neuville S., Esser N., Malaise D., Malaise O., Calvo F.Q., Relic B., Malaise M.G. (2013). Acute-phase serum amyloid a in osteoarthritis: Regulatory mechanism and proinflammatory properties. PLoS ONE.

[38] Ye H., Cai T., Shen Y., Zhao L., Zhang H., Yang J., Li F., Chen J., Shui X. (2024). MST1 knockdown inhibits osteoarthritis progression through Parkin-mediated mitophagy and Nrf2/NF-κB signalling pathway. J. Cell. Mol. Med..

[39] Kraus V.B., Reed A., Soderblom E.J., Golightly Y.M., Nelson A.E., Li Y.J. (2024). Serum proteomic biomarkers diagnostic of knee osteoarthritis. Osteoarthr. Cartil..

[40] Kim D., Kim J.E., Lee S.B., Lee N.Y., Park S.Y. (2024). Gulp1 regulates chondrocyte growth arrest and differentiation via the TGF-β/SMAD2/3 pathway. FEBS Lett..

[41] Myron Johnson A., Merlini G., Sheldon J., Ichihara K., Scientific Division Committee on Plasma Proteins (C-PP), International Federation of Clinical Chemistry and Laboratory Medicine (IFCC) (2007). Clinical indications for plasma protein assays: Transthyretin (prealbumin) in inflammation and malnutrition. Clin. Chem. Lab. Med..

[42] Wieczorek E., Ożyhar A. (2021). Transthyretin: From Structural Stability to Osteoarticular and Cardiovascular Diseases. Cells.

[43] Akasaki Y., Reixach N., Matsuzaki T., Alvarez-Garcia O., Olmer M., Iwamoto Y., Buxbaum J.N., Lotz M.K. (2015). Transthyretin deposition in articular cartilage: A novel mechanism in the pathogenesis of osteoarthritis. Arthritis Rheumatol..

[44] Matsuzaki T., Akasaki Y., Olmer M., Alvarez-Garcia O., Reixach N., Buxbaum J.N., Lotz M.K. (2017). Transthyretin deposition promotes progression of osteoarthritis. Aging Cell.

[45] Takinami Y., Yoshimatsu S., Uchiumi T., Toyosaki-Maeda T., Morita A., Ishihara T., Yamane S., Fukuda I., Okamoto H., Numata Y. (2013). Identification of potential prognostic markers for knee osteoarthritis by serum proteomic analysis. Biomark. Insights.

[46] Pei J., Wang G., Yang M., Liu L. (2025). Relaxin-2 counteracts TNF-α-induced senescence in human primary chondrocytes by enhancing telomerase activity and modulating SIRT1/p53 signaling. Peptides.

[47] Li C., Tu Y., Rong R., Zhang Z., Chen W., Long L., Zhang Y., Wang C., Pan B., Wu X. (2024). Association of thyroid hormone with osteoarthritis: From mendelian randomization and RNA sequencing analysis. J. Orthop. Surg. Res..

[48] Xu J.X., Xu F.Z., Furbish A., Braxton A.M., Brumfield B., Helke K.L., Peterson Y.K. (2024). Inhibition of complement C3 prevents osteoarthritis progression in guinea pigs by blocking STAT1 activation. Commun. Biol..

[49] Jiang Y., Ng C.Y., Seow D.S.K., Loh J.T., Wong R.C.W., Hui J.H.P., Toh W.S. (2026). Mesenchymal stem cell exosomes alleviate osteoarthritis by inhibiting complement activation via a CD59-dependent pathway. Arthritis Res. Ther..

[50] Kraus V.B., Reed A., Soderblom E.J., Moseley M.A., Hsueh M.F., Attur M.G., Samuels J., Abramson S.B., Li Y.J. (2023). Serum proteomic panel validated for prediction of knee osteoarthritis progression. Osteoarthr. Cartil. Open.

[51] Naili J.E., Ahmed A.S., Hedström M., Simonsen M.B., Broström E.W., Harris H.E., Végvári Á., Aulin C. (2024). Proteomic analysis reveals biomarkers associated with performance-based joint function and patient-reported outcomes in knee osteoarthritis. Osteoarthr. Cartil. Open.

[52] Zhang X., Ma S., Naz S.I., Soderblom E.J., Jain V., Aliferis C., Kraus V.B. (2024). Immune System-Related Plasma Pathogenic Extracellular Vesicle Subpopulations Predict Osteoarthritis Progression. Int. J. Mol. Sci..

[53] Dell’Isola A., Allan R., Smith S.L., Marreiros S.S., Steultjens M. (2016). Identification of clinical phenotypes in knee osteoarthritis: A systematic review of the literature. BMC Musculoskelet. Disord..

[54] Mobasheri A., Loeser R. (2024). Clinical phenotypes, molecular endotypes and theratypes in OA therapeutic development. Nat. Rev. Rheumatol..

[55] Giordano R., Capriotti C., Gerra M.C., Kappel A., Østgaard S.E., Dallabona C., Arendt-Nielsen L., Petersen K.K. (2024). A potential link between inflammatory profiles, clinical pain, pain catastrophizing and long-term outcomes after total knee arthroplasty surgery. Eur. J. Pain.

[56] Nanus D.E., Davis E.T., Jones S.W. (2024). Pre-Operative Adiposity and Synovial Fluid Inflammatory Biomarkers Provide a Predictive Model for Post-Operative Outcomes Following Total Joint Replacement Surgery in Osteoarthritis Patients. Osteology.

[57] Gregory C.D., Rimmer M.P. (2023). Extracellular vesicles arising from apoptosis: Forms, functions, and applications. J. Pathol..

[58] Morel O., Jesel L., Freyssinet J.M., Toti F. (2011). Cellular mechanisms underlying the formation of circulating microparticles. Arterioscler. Thromb. Vasc. Biol..

[59] Clarke E.J., Chabronova A., Peffers M.J. (2025). Extracellular vesicles in cartilage homeostasis, osteoarthritis, and biomarker discovery. Connect. Tissue Res..

[60] Borberg E., Pashko S., Koren V., Burstein L., Patolsky F. (2021). Depletion of Highly Abundant Protein Species from Biosamples by the Use of a Branched Silicon Nanopillar On-Chip Platform. Anal. Chem..

[61] Styrkarsdottir U., Lund S.H., Saevarsdottir S., Magnusson M.I., Gunnarsdottir K., Norddahl G.L., Frigge M.L., Ivarsdottir E.V., Bjornsdottir G., Holm H. (2021). The CRTAC1 Protein in Plasma Is Associated With Osteoarthritis and Predicts Progression to Joint Replacement: A Large-Scale Proteomics Scan in Iceland. Arthritis Rheumatol..

[62] Szilagyi I.A., Vallerga C.L., Boer C.G., Schiphof D., Ikram M.A., Bierma-Zeinstra S.M.A., van Meurs J.B.J. (2023). Plasma proteomics identifies CRTAC1 as a biomarker for osteoarthritis severity and progression. Rheumatology.

[63] Kang Z., Zhang J., Liu W., Zhu C., Zhu Y., Li P., Li K., Tong Q., Dai S.M. (2025). Plasma Proteomic Profiles Predict Individual Future Osteoarthritis Risk. Arthritis Rheumatol..

[64] Clark A.G., Jordan J.M., Vilim V., Renner J.B., Dragomir A.D., Luta G., Kraus V.B. (1999). Serum cartilage oligomeric matrix protein reflects osteoarthritis presence and severity: The Johnston County Osteoarthritis Project. Arthritis Rheumatol..

[65] Tseng S., Reddi A.H., Di Cesare P.E. (2009). Cartilage Oligomeric Matrix Protein (COMP): A Biomarker of Arthritis. Biomark. Insights.

[66] Cui J., Zhang J. (2022). Cartilage Oligomeric Matrix Protein, Diseases, and Therapeutic Opportunities. Int. J. Mol. Sci..

[67] Beker M.C., Caglayan B., Yalcin E., Caglayan A.B., Turkseven S., Gurel B., Kelestemur T., Sertel E., Sahin Z., Kutlu S. (2018). Time-of-Day Dependent Neuronal Injury After Ischemic Stroke: Implication of Circadian Clock Transcriptional Factor Bmal1 and Survival Kinase AKT. Mol. Neurobiol..

[68] Cevik O., Baykal A.T., Sener A. (2016). Platelets Proteomic Profiles of Acute Ischemic Stroke Patients. PLoS ONE.

[69] Tang D., Chen M., Huang X., Zhang G., Zeng L., Zhang G., Wu S., Wang Y. (2023). SRplot: A free online platform for data visualization and graphing. PLoS ONE.

[70] Medina I., Carbonell J., Pulido L., Madeira S.C., Goetz S., Conesa A., Tárraga J., Pascual-Montano A., Nogales-Cadenas R., Santoyo J. (2010). Babelomics: An integrative platform for the analysis of transcriptomics, proteomics and genomic data with advanced functional profiling. Nucleic Acids Res..

[71] Gentleman R.C., Carey V.J., Bates D.M., Bolstad B., Dettling M., Dudoit S., Ellis B., Gautier L., Ge Y., Gentry J. (2004). Bioconductor: Open software development for computational biology and bioinformatics. Genome Biol..

[72] Huber W., Carey V.J., Gentleman R., Anders S., Carlson M., Carvalho B.S., Bravo H.C., Davis S., Gatto L., Girke T. (2015). Orchestrating high-throughput genomic analysis with Bioconductor. Nat. Methods.

