# Supplementary material for: Plasma Proteomics Reveals Persistent and Surgery-Responsive Molecular Signatures in Osteoarthritis Patients
Source: Int J Mol Sci. 2026 Mar 21;27(6):2862. doi: 10.3390/ijms27062862 (PMC13027247; doi:10.3390/ijms27062862)
Supplement: Supplementary file 1 [file ijms-27-02862-s001.zip › ijms-4174388-supplementary.pdf]

Article Title: Plasma Proteomics Reveals Persistent and Surgery-Responsive Molecular Signatures in Osteoarthritis Patients

Journal: International Journal of Molecular Sciences

Authors: Duygu Sari-Ak*, Fatih Con, Melike Guvendi, Hayriye E. Yelkenci, Nazli Helvaci, Alev Kural, Marcel Zamocky, Cemal Kural and Mustafa C. Beker*

* Corresponding author at: Department of Medical Biology, Hamidiye International School of Medicine, University of Health Sciences, Istanbul 34668, Türkiye.
  E-mail address: duygu.sariak@sbu.edu.tr; Tel.: +90 533 511 14 60

* Corresponding author at: Department of Physiology, School of Medicine, Istanbul Medeniyet University, Istanbul 34668, Türkiye.
  E-mail address: mustafa.beker@medeniyet.edu.tr; Tel: +90 535 888 85 01

Table S1 Protein Abbreviations and Full Names Corresponding to Proteins Detected in Osteoarthritis Study. This table provides the full names of protein abbreviations used in the study, which are detected in osteoarthritis patients through proteomic analysis. The abbreviations correspond to protein identifiers found in the Human Protein Atlas database, accessible at https://www.proteinatlas.org . These proteins play crucial roles in various biological processes such as inflammation, extracellular matrix remodeling, and metabolic regulation, which are implicated in osteoarthritis pathogenesis and recovery.

Abbreviation	Full Name
ACOXL	Acyl-CoA Oxidase-Like
ACSL5	Acyl-CoA Synthetase Long Chain Family Member 5
ACTB	Actin Beta
ACTBL2	Actin, Beta-Like 2
ADCY8	Adenylate Cyclase 8
ADRA2C	Adrenoceptor Alpha 2C
AFF3	AF4/FMR2 Family Member 3
AFG3L2	AFG3 Like Matrix AAA Peptidase Subunit 2
AFM	Afamin
AGAP6	ArfGAP with GTPase Domain, Ankyrin Repeat and PH Domain 6
AHSG	Alpha-2-HS-Glycoprotein
AKAP10	A-Kinase Anchoring Protein 10
ALB	Albumin
ALDOA	Aldolase A, Fructose-Bisphosphate
AMBP	Alpha-1-Microglobulin/Bikunin Precursor
AMPD3	Adenosine Monophosphate Deaminase 3
ANKRD26	Ankyrin Repeat Domain 26
APOA1	Apolipoprotein A1
APOA2	Apolipoprotein A2
APOC4	Apolipoprotein C4
APOC4-APOC2	Apolipoprotein C4 and Apolipoprotein C2
APOD	Apolipoprotein D
APOH	Apolipoprotein H (Beta-2-Glycoprotein I)
APOL1	Apolipoprotein L1
APOM	Apolipoprotein M
ARG1	Arginase 1
ATP10A	ATPase Phospholipid Transporting 10A
ATP6V0D1	ATPase H+ Transporting V0 Subunit D1
ATG13	Autophagy Related 13

AZGP1	Alpha-2-Glycoprotein 1
B2M	Beta-2-Microglobulin
BBS9	Bardet-Biedl Syndrome 9
BIN3	Bridging Integrator 3
BNC2	Basonuclin 2
C1QB	Complement Component 1, Q Subcomponent, B Chain
C1QTNF6	C1q and TNF Related 6
C1R	Complement Component 1r
C4BPA	Complement Component 4 Binding Protein Alpha
C4-APOC2	Apolipoprotein C-II
C6	Complement Component 6
C8A	Complement component C8 alpha chain
C9	Complement component C9
CADPS	Calcium Dependent Secretion Activator
CADPS2	Calcium Dependent Secretion Activator 2
CAMP	Cathelicidin Antimicrobial Peptide
CASP5	Caspase 5
CAPN11	Calpain 11
CARMIL3	Capping Protein Regulator and Myosin 1 Linker 3
CCDC150	Coiled-Coil Domain Containing 150
CETP	Cholesteryl Ester Transfer Protein
CD5L	CD5 Molecule Like
CDH5	Cadherin 5
CDH11	Cadherin 11
CEP350	Centrosome-associated protein 350
CETP	Cholesteryl ester transfer protein
CFHR1	Complement Factor H Related 1
CFHR2	Complement Factor H Related 2
CFHR5	Complement Factor H Related 5
CHPF	Chondroitin Polymerizing Factor
CHTF18	Chromosome Transmission Fidelity Factor 18
CHN2	Chimerin 2
CLIC5	Chloride Intracellular Channel 5
CLIP1	CAP-Gly Domain Containing Linker Protein 1

CLU	Clusterin
CNTNAP3B	Contactin-associated protein-like 3B
CO4A3	Collagen Type IV Alpha 3 Chain
COG5	Component of Oligomeric Golgi Complex 5
COL4A3	Collagen Type IV Alpha 3 Chain
COL6A1	Collagen Type VI Alpha 1 Chain
COMP	Cartilage Oligomeric Matrix Protein
COPS6	COP9 Signalosome Subunit 6
CP	Ceruloplasmin
CPN2	Carboxypeptidase N Subunit 2
CRISP3	Cysteine-Rich Secretory Protein 3
CST3	Cystatin C
CTHRC1	Collagen Triple Helix Repeat Containing 1
CTSD	Cathepsin D
CXCL4V1	Platelet Factor 4 Variant 1
DBH	Dopamine Beta-Hydroxylase
DDB1	Damage Specific DNA Binding Protein 1
DDX3X	DEAD-box Helicase 3 X-linked
DHRS11	Dehydrogenase/reductase 11
DOCK10	Dedicator of Cytokinesis 10
DYNC1I2	Dynein Cytoplasmic 1 Intermediate Chain 2
EDEM3	ER Degradation Enhancing Alpha-Mannosidase Like Protein 3
EGLN2	Egl-9 Family Hypoxia Inducible Factor 2
EML5	Echinoderm Microtubule Associated Protein-Like 5
EPDR1	Ependymin Related 1
EZR	Ezrin
F5	Coagulation factor V
F9	Coagulation factor IX
F10	Coagulation Factor X
F11	Coagulation Factor XI
F13B	Coagulation Factor XIII B Chain
FANCA	Fanconi Anemia Complementation Group A Protein
FANCM	Fanconi Anemia Complementation Group M Protein
FBLN1	Fibulin-1

FCN3	Ficolin 3
FEN1	Flap Structure-specific Endonuclease 1
FETUB	Fetuin B
FERMT3	Fermitin family homolog 3
FGA	Fibrinogen Alpha Chain
FGB	Fibrinogen Beta Chain
FGD1	FYVE, RhoGEF and PH domain-containing protein1
FGFR2	Fibroblast Growth Factor Receptor 2
FOXP2	Forkhead Box P2
GAS7	Growth Arrest Specific 7
GC	Group-Specific Component (Vitamin D Binding Protein)
GALC	Galactosylceramidase
GLYATL1	Glycine N-Acyltransferase Like 1
GOLGB1	Golgin B1
GOLPH3	Golgi Phosphoprotein 3
GP5	Glycoprotein V Platelet
GPLD1	Glycosylphosphatidylinositol Specific Phospholipase D1
GPX3	Glutathione Peroxidase 3
GRID1	Glutamate Receptor Ionotropic, Delta 1
GSN	Gelsolin
GTF2E1	General Transcription Factor IIE Subunit 1
HABP2	Hyaluronan Binding Protein 2
HEMK1	HemK Methyltransferase Family Member 1
HERC3	HECT and RLD Domain Containing E3 Ubiquitin Protein Ligase 3
HERC4	HECT and RLD Domain Containing E3 Ubiquitin Protein Ligase 4
HGFAC	Hepatocyte Growth Factor Activator
HOOK1	Hook Microtubule Tethering Protein 1
HOXC9	Homeobox C9
HP	Haptoglobin
HPR	Haptoglobin-Related Protein
HPX	Hemopexin
HYPK	Huntingtin Interacting Protein K
IGFBP3	Insulin-Like Growth Factor Binding Protein 3

IGFALS	Insulin-Like Growth Factor Binding Protein, Acid Labile Subunit
IGHA2	Immunoglobulin Heavy Constant Alpha 2
IGHG1	Immunoglobulin Heavy Constant Gamma 1
IGHG3	Immunoglobulin Heavy Constant Gamma 3
IFT122	Intraflagellar Transport 122
IFT172	Intraflagellar Transport 172
IL1RAP	Interleukin 1 Receptor Accessory Protein
INPPL1	Inositol Polyphosphate Phosphatase like 1
INTS6	Integrator Complex Subunit 6
IRF7	Interferon Regulatory Factor 7
IST1	IST1 Homolog (IST1 Factor of Spastin, IST1 Factor of ESCRT Complex)
ITIH2	Inter-Alpha-Trypsin Inhibitor Heavy Chain H2
ITIH3	Inter-Alpha-Trypsin Inhibitor Heavy Chain H3
ITIH4	Inter-Alpha-Trypsin Inhibitor Heavy Chain H4
JADE1	Jade Family PHD Finger 1
KIF16B	Kinesin Family Member 16B
KLK15	Kallikrein Related Peptidase 15
KNG1	Kininogen 1
LBP	Lipopolysaccharide Binding Protein
LCAT	Lecithin-Cholesterol Acyltransferase
LCP1	Lymphocyte Cytosolic Protein 1
LDHB	Lactate Dehydrogenase B Chain
LRRC9	Leucine-Rich Repeat Containing 9
MAST1	Microtubule Associated Serine/Threonine Kinase 1
MASP1	Mannan Binding Lectin Serine Protease 1
METTL4	Methyltransferase Like 4
MFN2	Mitofusin 2
MMP2	Matrix Metallopeptidase 2
MMP19	Matrix Metallopeptidase 19
MST1	Macrophage Stimulating 1
NADK2	NAD Kinase 2, Mitochondrial
NGLY1	N-glycanase 1
NOVA1	Nova Alternative Splicing Regulator 1
NPAS3	Neuronal PAS Domain Protein 3

NSD1	Nuclear Receptor Binding SET Domain Protein 1
NT5C1B	Cytosolic 5'-nucleotidase 1B
OBSCN	Obscurin
OFD1	OFD1 Centriole and Centriolar Satellite Protein
ORM1	Orosomucoid 1
ORM2	Orosomucoid 2
PBXIP1	PBX Homeobox Interacting Protein 1
PDE4DIP	Phosphodiesterase 4D Interacting Protein
PKM	Pyruvate Kinase, Muscle
PLG	Plasminogen
PLCB1	Phospholipase C Beta 1
POLRMT	DNA Directed RNA Polymerase Mitochondrial
POMT1	Protein O-Mannosyltransferase 1
POSTN	Periostin
PPBP	Pro-Platelet Basic Protein
PPM1A	Protein Phosphatase, Mg2+/Mn2+ Dependent 1A
PPP1R14A	Protein Phosphatase 1 Regulatory Inhibitor Subunit 14A
PPP3R2	Protein Phosphatase 3 Regulatory Subunit Beta
PROC	Protein C, Inactivator of Coagulation Factors Va and VIIIa
PZP	Pregnancy zone protein
R3HCC1	R3H Domain Containing 1
RAB34	RAB34, Member RAS Oncogene Family
RBBP7	Retinoblastoma Binding Protein 7
RBP4	Retinol Binding Protein 4
RCN1	Reticulocalbin 1
REST	RE1 Silencing Transcription Factor
RIGI	Retinoic Acid Inducible Gene I
RPS6KC1	Ribosomal Protein S6 Kinase C1
S100A9	S100 Calcium Binding Protein A9
SAA1	Serum amyloid A1
SBF2	SET Binding Factor 2
SERPINA1	Serpin Family A Member 1
SERPINA3	Serpin Family A Member 3
SERPINA4	Serpin Family A Member 4

SERPINC1	Serpin Family C Member 1
SERPINI1	Serpin Family I Member 1
SERPINF2	Serpin Family F Member 2
SETD5	Histone-lysine N-methyltransferase SETD5
SLC3A2	Solute Carrier Family 3 Member 2
SNAPC3	snRNA-activating protein complex subunit 3
SNX6	Sorting Nexin 6
SPEF2	Sperm Flagellar 2
SPP2	Secreted Phosphoprotein 2
STAB1	Stabilin 1
STAU2	Staufen Double-Stranded RNA Binding Protein 2
TAGLN2	Transgelin 2
TENT5D	Terminal Nucleotidyltransferase 5D
TF	Transferrin
TGFBI	Transforming Growth Factor Beta-Induced Protein
THBS1	Thrombospondin 1
TOM1	Target of Myb1 Membrane Trafficking Protein
TPI1	Triosephosphate Isomerase 1
TRIM15	Tripartite Motif Containing 15
TRIM27	Tripartite Motif Containing 27
TRIM52	Tripartite Motif Containing 52
TTLL11	Tubulin Tyrosine Ligase Like 11
TTR	Transthyretin
UBE2U	Ubiquitin Conjugating Enzyme E2 U
USP13	Ubiquitin Specific Peptidase 13
VCAM1	Vascular Cell Adhesion Molecule 1
VNN1	Vanin 1
VTN	Vitronectin
VWF	Von Willebrand Factor
WDR41	WD repeat domain 41
YWHAQ	Tyrosine 3-Monooxygenase/Tryptophan 5-Monooxygenase Activation Protein Q
YWHAZ	Tyrosine 3-Monooxygenase/Tryptophan 5-Monooxygenase Activation Protein Z
ZNF467	Zinc Finger Protein 467
ZNF468	Zinc Finger Protein 468

ZNF546	Zinc Finger Protein 546
ZNF624	Zinc Finger Protein 624

Table S2 Enrichment Analysis of Proteins in Pre-Operative Osteoarthritis Compared to Healthy Controls. This table presents the results of the enrichment analysis for proteins identified in the pre-operative osteoarthritis (OA) group compared to healthy controls. The table includes gene counts, the genes involved in each pathway, fold enrichment values, and the corresponding p-values, highlighting key pathways associated with OA-related molecular alterations.

Term	Gene count	Genes in each pathway from our result	Fold Enrichment	P-value
Complement and coagulation cascades	5	PROC, F10, CFHR1, C1R, F13B	10.3	1.20E-03
Relaxin signaling pathway	5	ACTA2, MMP2, COL4A3, ADCY8, PLCB1	7	5.10E-03
Thyroid hormone synthesis	4	TTR, ALB, ADCY8, PLCB1	9.7	7.50E-03
Gastric acid secretion	4	ADCY8, PLCB1, EZR, ACTB	9.6	7.80E-03
Glucagon signaling pathway	4	LDHB, PPP3R2, PDHB, PLCB1	6.8	2.00E-02
Platelet activation	4	ADCY8, PLCB1, GP5, ACTB	5.8	3.00E-02
Estrogen signaling pathway	4	MMP2, ADCY8, PLCB1, CTSD	5.2	3.90E-02
Long-term potentiation	3	PPP3R2, ADCY8, PLCB1	8.1	5.00E-02
Platelet activation, signaling and aggregation	11	SPARC, AHSG, APOH, ALB, TAGLN2, PPBP, ADRA2C, ORM2, YWHAZ, GP5, SERPINA4	6.7	4.30E-06
Platelet degranulation	8	SPARC, AHSG, APOH, ALB, TAGLN2, PPBP, ORM2, SERPINA4	9.9	1.40E-05
Hemostasis	16	SPARC, F10, AHSG, PPBP, ADRA2C, ORM2, YWHAZ, GP5, SERPINA4, ACTB, PROC, APOH, ALB, MFN2, F13B, TAGLN2	3.7	1.60E-05
Response to elevated platelet cytosolic Ca2+	8	SPARC, AHSG, APOH, ALB, TAGLN2, PPBP, ORM2, SERPINA4	9.5	1.80E-05
Extracellular matrix organization	11	ACTA2, CAPN11, SPARC, TTR, MMP2, COL4A3, COL6A1, MMP19, FBLN1, CTSD, ACTB	5.5	2.40E-05
Neutrophil degranulation	13	DDX3X, AHSG, IST1, HP, PPBP, HPR, ORM2, VNN1, TTR, TOM1, CTSD, B2M, CAMP	4.3	3.50E-05
Binding and Uptake of Ligands by Scavenger Receptors	6	SPARC, AMBP, ALB, STAB1, HP, HPR	9.1	4.80E-04
Collagen degradation	5	MMP2, COL4A3, COL6A1, MMP19, CTSD	12.6	6.10E-04
Innate Immune System	17	DDX3X, AHSG, IST1, C1R, HP, PPBP, HPR, ORM2, ACTB, VNN1, TTR, CFHR1,<br>TOM1, ATP6V0D1, CTSD, B2M, CAMP	2.4	1.20E-03
Degradation of the extracellular matrix	6	CAPN11, MMP2, COL4A3, COL6A1, MMP19, CTSD	6.9	1.70E-03
Formation of Fibrin Clot (Clotting Cascade)	4	PROC, F10, F13B, GP5	15.8	2.00E-03

Regulation of Insulin-like Growth Factor (IGF) transport and uptake by Insulin-like Growth Factor Binding Proteins (IGFBPs)	5	RCN1, PROC, AHSG, MMP2, ALB	6.4	7.40E-03
Common Pathway of Fibrin Clot Formation	3	PROC, F10, F13B	21.1	8.50E-03
Intrinsic Pathway of Fibrin Clot Formation	3	PROC, F10, GP5	19.4	1.00E-02
Scavenging of heme from plasma	4	AMBP, ALB, HP, HPR	8.4	1.10E-02
Non-integrin membrane-ECM interactions	4	ACTA2, TTR, COL4A3, ACTB	8	1.30E-02
Defective visual phototransduction due to STRA6 loss of function	2	RBP4, TTR	107.9	1.80E-02
Scavenging by Class H Receptors	2	SPARC, STAB1	80.9	2.40E-02
Post-translational protein phosphorylation	4	RCN1, PROC, AHSG, ALB	5.9	2.90E-02
EPH-ephrin mediated repulsion of cells	3	EPHA10, MMP2, ACTB	9.7	3.70E-02
Signaling by Receptor Tyrosine Kinases	8	REST, SPARC, COL4A3, MST1, COL6A1, ATP6V0D1, CTSD, ACTB	2.4	4.50E-02
proteolysis	12	USP13, CAPN11, PROC, F10, C1R, MMP2, MST1, HP, MMP19, KLK15, HPR, CTSD	4.4	8.10E-05
acute-phase response	4	AHSG, HP, HPR, ORM2	22.4	7.30E-04
postsynaptic actin cytoskeleton organization	3	ACTBL2, EZR, ACTB	35.4	3.10E-03
zymogen activation	3	C1R, HP, HPR	28	5.00E-03
extracellular matrix organization	5	MMP2, COL4A3, POMT1, MMP19, FBLN1	6.8	6.00E-03
defense response to bacterium	5	STAB1, HP, PPBP, HPR, CAMP	6.5	7.00E-03
blood coagulation	4	PROC, F10, F13B, GP5	8.7	1.10E-02
cell adhesion	8	AZGP1, AMBP, CDH11, COL4A3, COL6A1, STAB1, PBXIP1, GP5	3.2	1.20E-02
response to hydrogen peroxide	3	MMP2, HP, HPR	17.7	1.20E-02
negative regulation of hydrogen peroxide catabolic process	2	HP, HPR	149.3	1.30E-02
embryo implantation	3	MMP2, MST1, FBLN1	14.9	1.70E-02
extracellular matrix disassembly	3	MMP2, MMP19, PBXIP1	14.3	1.80E-02
negative regulation of angiogenesis	4	SPARC, APOH, COL4A3, STAB1	6.7	2.20E-02
vitamin transport	2	AFM, GC	89.6	2.20E-02
blood coagulation, fibrin clot formation	2	FBLN1, F13B	64	3.10E-02
cell migration	5	GOLPH3, CARMIL3, MMP2, CDH11, PBXIP1	4.1	3.20E-02
blood coagulation, intrinsic pathway	2	APOH, GP5	56	3.50E-02

ovulation from ovarian follicle	2	MMP2, MMP19	49.8	3.90E-02
negative regulation of oxidoreductase activity	2	MMP2, MMP19	49.8	3.90E-02
chronic inflammatory response	2	VNN1, CAMP	44.8	4.30E-02
cellular oxidant detoxification	3	AMBP, ALB, HP	9	4.30E-02
insulin-like growth factor receptor signaling pathway	3	EPHA10, COL6A1, PLCB1	8.3	5.00E-02
defense response	3	HP, PPBP, HPR	8.3	5.00E-02
extracellular space	38	SPARC, C1R, PZP, HP, AFM, LCAT, FBLN1, HPR, ORM2, SERPINA4, ACTB, TTR, C1QTNF6, APOH, GC, CTSD, B2M, CAMP, AMBP, F10, AHSG, MMP2, MST1, KLK15, PPBP, YWHAZ, ACTA2, AZGP1, RBP4, PROC, ACTBL2, CFHR1, COL4A3, ALB, MMP19, F13B, SERPINI1, EZR	4.8	1.00E-16
extracellular region	40	SPARC, SPEF2, DDX3X, EPHA10, C1R, PZP, HP, AFM, LCAT, FBLN1, HPR, ORM2, SERPINA4, TTR, C1QTNF6, APOH, GC, CTSD, B2M, CAMP, OFD1, AMBP, F10, AHSG, IST1, MMP2, MST1, PPBP, AZGP1, RBP4, VNN1, PROC, CFHR1, COL4A3, COL6A1, ALB, CDH11, MMP19, TAGLN2, F13B	4	2.80E-15
extracellular exosome	37	DDX3X, C1R, PZP, HP, AFM, LCAT, FBLN1, HPR, ORM2, SERPINA4, ACTB, LDHB, TTR, APOH, TOM1, GC, CTSD, B2M, CAMP, AMBP, AHSG, IST1, YWHAZ, GP5, ACTA2, EML5, AZGP1, RBP4, ACTBL2, COL6A1, ALB, CDH11, TAGLN2, SERPINI1, PLCB1, EZR, ATP6V0D1	3.9	2.50E-13
blood microparticle	13	AMBP, AHSG, C1R, PZP, HP, AFM, HPR, ORM2, YWHAZ, ACTB, CFHR1, ALB, GC	20.5	1.40E-12
collagen-containing extracellular matrix	13	SPARC, AMBP, AHSG, MMP2, PZP, MST1, FBLN1, ORM2, AZGP1, APOH, COL4A3, COL6A1, CTSD	7.8	8.40E-08
tertiary granule lumen	6	HP, PPBP, HPR, CTSD, B2M, CAMP	25	3.90E-06
specific granule lumen	6	HP, HPR, CTSD, B2M, ORM2, CAMP	22.3	7.00E-06
platelet alpha granule lumen	5	SPARC, AHSG, ALB, PPBP, ORM2	17.2	2.00E-04
endoplasmic reticulum lumen	8	RCN1, PROC, F10, AHSG, COL4A3, ALB, COL6A1, B2M	5.9	4.00E-04
basement membrane	5	ACTA2, SPARC, COL4A3, COL6A1, FBLN1	12	7.70E-04
actin cytoskeleton	7	ACTA2, ACTBL2, TAGLN2, INTS6, ADCY8, EZR, ACTB	5.6	1.50E-03
endocytic vesicle lumen	3	SPARC, HP, HPR	35	3.20E-03
vesicle	5	TAGLN2, EZR, YWHAZ, CAMP, ACTB	7.1	5.20E-03
dense body	2	ACTBL2, ACTB	66.8	2.90E-02
glutamatergic synapse	7	CARMIL3, ACTBL2, CDH11, ADCY8, PLCB1, YWHAZ, ACTB	2.8	3.90E-02

oxidoreductase complex	2	LDHB, METTL4	46.7	4.20E-02
endosome	5	GOLPH3, TOM1, EZR, ADRA2C, ATP6V0D1	3.8	4.20E-02
lamellipodium	4	ACTA2, DDX3X, CARMIL3, ACTB	4.9	4.70E-02
serine-type endopeptidase activity	9	PROC, F10, C1R, MMP2, MST1, HP, MMP19, KLK15, HPR	10.3	2.50E-06
antioxidant activity	4	AMBP, ALB, HP, HPR	41.7	1.10E-04
calcium ion binding	11	CN1, CAPN11, SPARC, PROC, PPP3R2, F10, C1R, CDH11, STAB1, FBLN1, PLCB1	3.3	1.80E-03
protein binding	73	SPARC, DDX3X, CHPF, NGLY1, POMT1, HP, WDR41, CHTF18, ORM2, SERPINA4, ACTB, HERC3, C1QTNF6, STAB1, TOM1, BBS9, B2M, CTSD, CAMP, OFD1, HEMK1, AHSG, IST1, MMP2, MST1, ADRA2C, ATG13, YWHAZ, TENT5D, ACTA2, AZGP1, RBP4, RCN1, ACTBL2, COL4A3, COL6A1, TAGLN2, EZR, ATP6V0D1, PLCB1, CCDC150, USP13, EPHA10, C1R, AFM, LCAT, FBLN1, ATP10A, PBXIP1, PDHB, GTF2E1, LDHB, PPP3R2, TTR, NSD1, APOH, MFN2, ZNF624, HOXC9, ZNF467, ANKRD26, AMBP, F10, KLK15, PPBP, GP5, GOLPH3, REST, PROC, CFHR1, GOLGB1, ALB, INTS6	1.2	8.80E-03
identical protein binding	16	OFD1, IST1, C1R, FBLN1, YWHAZ, ACTB, LDHB, REST, TTR, CFHR1, C1QTNF6, APOH, ALB, PLCB1, EZR, B2M	2	1.30E-02
serine-type endopeptidase inhibitor activity	4	AMBP, PZP, SERPINI1, SERPINA4	8	1.40E-02
cadherin binding	6	DDX3X, IST1, CDH11, TAGLN2, EZR, YWHAZ	4.1	1.50E-02
hemoglobin binding	2	HP, HPR	87.5	2.20E-02
serine-type peptidase activity	3	PROC, C1R, KLK15	9.7	3.80E-02

Table S3 Enrichment Analysis of Proteins in Post-Operative Osteoarthritis Compared to Healthy Controls. This table presents the results of the enrichment analysis for proteins identified in the post-operative osteoarthritis (OA) group compared to healthy controls. The table includes gene counts, the genes involved in each pathway, fold enrichment values, and the corresponding p-values, highlighting key pathways associated with OA-related molecular alterations.

Term	Gene count	Genes in each pathway from our result	Fold Enrichment	P-value
Complement and coagulation cascades	7	FGB, PROC, F10, CFHR1, C1R, SERPINC1, F13B	12.1	2.00E-05
Platelet activation	5	FGB, ADCY8, PLCB1, GP5, ACTB	6	8.60E-03
Relaxin signaling pathway	5	ACTA2, MMP2, COL4A3, ADCY8, PLCB1	5.9	9.60E-03
Thyroid hormone synthesis	4	TTR, ALB, ADCY8, PLCB1	8.1	1.20E-02
Gastric acid secretion	4	ADCY8, PLCB1, EZR, ACTB	8	1.30E-02
Hemostasis	19	FGB, SPARC, F10, AHSG, SERPINC1, PPBP, ADRA2C, ORM2, YWHAZ, GP5, SERPINA4, ACTB, PROC, APOH, ALB, MFN2, F13B, TAGLN2, IGKV3-11	3.8	1.30E-06
Platelet activation, signaling and aggregation	12	FGB, SPARC, AHSG, APOH, ALB, TAGLN2, PPBP, ADRA2C, ORM2, YWHAZ, GP5, SERPINA4	6.3	2.40E-06
Platelet degranulation	9	FGB, SPARC, AHSG, APOH, ALB, TAGLN2, PPBP, ORM2, SERPINA4	9.6	3.70E-06
Response to elevated platelet cytosolic Ca2+	9	FGB, SPARC, AHSG, APOH, ALB, TAGLN2, PPBP, ORM2, SERPINA4	9.3	4.90E-06
Neutrophil degranulation	15	DDX3X, AHSG, IST1, ARG1, HP, PPBP, HPR, IQGAP2, ORM2, TTR, VNN1, TOM1, CTSD, B2M, CAMP	4.3	7.20E-06
Formation of Fibrin Clot (Clotting Cascade)	6	FGB, PROC, F10, SERPINC1, F13B, GP5	20.5	9.40E-06
Innate Immune System	23	FGB, DDX3X, AHSG, IST1, C1R, ARG1, HP, PPBP, HPR, IQGAP2, ORM2, ACTB, IGHG3, TTR, VNN1, CFHR1, SAA1, TOM1, IGKV3-11, ATP6V0D1, CTSD, B2M, CAMP	2.8	9.50E-06
Common Pathway of Fibrin Clot Formation	5	FGB, PROC, F10, SERPINC1, F13B	30.5	1.80E-05
Extracellular matrix organization	11	FGB, ACTA2, SPARC, TTR, MMP2, COL4A3, COL6A1, MMP19, FBLN1, CTSD, ACTB	4.8	8.60E-05
Binding and Uptake of Ligands by Scavenger Receptors	7	SPARC, AMBP, ALB, SAA1, HP, HPR, IGKV3-11	9.2	1.00E-04
Intrinsic Pathway of Fibrin Clot Formation	4	PROC, F10, SERPINC1, GP5	22.4	6.90E-04
Collagen degradation	5	MMP2, COL4A3, COL6A1, MMP19, CTSD	11	1.10E-03
Regulation of Insulin-like Growth Factor (IGF) transport and uptake by Insulin-like Growth Factor Binding Proteins (IGFBPs)	6	RCN1, PROC, AHSG, SERPINC1, MMP2, ALB	6.6	2.00E-03

Scavenging of heme from plasma	5	AMBP, ALB, HP, HPR, IGKV3-11	9.1	2.10E-03
Immune System	27	DDX3X, C1R, HP, HPR, IQGAP2, ORM2, ACTB, IGHG3, HERC3, TTR, TOM1, IGKV3-11, CTSD, B2M, CAMP, FGB, FANCM, AHSG, IST1, ARG1, MMP2, PPBP, YWHAZ, VNN1, CFHR1, SAA1, ATP6V0D1	1.7	3.20E-03
Post-translational protein phosphorylation	5	RCN1, PROC, AHSG, SERPINC1, ALB	6.4	7.50E-03
Degradation of the extracellular matrix	5	MMP2, COL4A3, COL6A1, MMP19, CTSD	5	1.70E-02
Non-integrin membrane-ECM interactions	4	ACTA2, TTR, COL4A3, ACTB	6.9	1.90E-02
Defective visual phototransduction due to STRA6 loss of function	2	RBP4, TTR	93.4	2.10E-02
Platelet Aggregation (Plug Formation)	3	FGB, ADRA2C, GP5	10.8	3.10E-02
Regulation of Complement cascade	4	IGHG3, CFHR1, C1R, IGKV3-11	4.9	4.60E-02
EPH-ephrin mediated repulsion of cells	3	EPHA10, MMP2, ACTB	8.4	4.80E-02
acute-phase response	5	AHSG, SAA1, HP, HPR, ORM2	24.8	4.60E-05
blood coagulation	6	FGB, PROC, F10, SERPINC1, F13B, GP5	11.6	1.60E-04
proteolysis	12	USP13, PROC, CASP5, F10, C1R, MMP2, MST1, HP, MMP19, KLK15, HPR, CTSD	3.9	2.40E-04
blood coagulation, fibrin clot formation	3	FGB, FBLN1, F13B	85.2	5.10E-04
zymogen activation	3	C1R, HP, HPR	24.8	6.30E-03
extracellular matrix organization	5	MMP2, COL4A3, POMT1, MMP19, FBLN1	6.1	9.20E-03
memory	4	FEN1, DBH, ADCY8, PLCB1	9.1	9.40E-03
negative regulation of hydrogen peroxide catabolic process	2	HP, HPR	132.5	1.50E-02
response to hydrogen peroxide	3	MMP2, HP, HPR	15.7	1.50E-02
embryo implantation	3	MMP2, MST1, FBLN1	13.3	2.10E-02
extracellular matrix disassembly	3	MMP2, MMP19, PBXIP1	12.7	2.30E-02
innate immune response	8	FGB, TRIM52, DDX3X, VNN1, C1R, ARG1, B2M, CAMP	2.8	2.40E-02
regulation of extrinsic apoptotic signaling pathway	2	ACSL5, DBH	79.5	2.50E-02
cellular response to interleukin-1	3	FGB, MMP2, CAMP	10.8	3.10E-02
blood coagulation, intrinsic pathway	2	APOH, GP5	49.7	3.90E-02
platelet activation	3	FGB, SAA1, ADRA2C	9	4.30E-02

negative regulation of oxidoreductase activity	2	HP, HPR	44.2	4.40E-02
ovulation from ovarian follicle	2	MMP2, MMP19	44.2	4.40E-02
cell migration	5	GOLPH3, CARMIL3, MMP2, CDH11, PBXIP1	3.7	4.70E-02
chronic inflammatory response	2	VNN1, CAMP	39.8	4.90E-02
extracellular region	48	SPARC, DHRS11, SPEF2, DDX3X, EPHA10, C1R, SERPINC1, PZP, HP, LCAT, FBLN1, DBH, HPR, ORM2, SERPINA4, IGHG3, TTR, C1QTNF6, APOH, IGKV2-24, GC, IGKV3-11, CTSD, B2M, CAMP, FGB, OFD1, AMBP, F10, AHSG, IST1, ARG1, MMP2, MST1, PPBP, AZGP1, RBP4, VNN1, PROC, CFHR1, COL4A3, COL6A1, ALB, CDH11, SAA1, MMP19, TAGLN2, F13B	4.3	1.80E-19
extracellular space	41	SPARC, C1R, SERPINC1, PZP, HP, LCAT, FBLN1, DBH, HPR, ORM2, SERPINA4, ACTB, IGHG3, TTR, C1QTNF6, APOH, GC, CTSD, B2M, CAMP, FGB, AMBP, F10, AHSG, ARG1, MMP2, MST1, KLK15, PPBP, YWHAZ, ACTA2, AZGP1, RBP4, PROC, CFHR1, COL4A3, ALB, MMP19, F13B, SERPINI1, EZR	4.5	4.90E-17
blood microparticle	16	FGB, AMBP, AHSG, C1R, SERPINC1, PZP, HP, HPR, ORM2, YWHAZ, ACTB, IGHG3, CFHR1, ALB, GC, IGKV3-11	22.3	4.30E-16
extracellular exosome	42	HBS1L, DDX3X, C1R, SERPINC1, PZP, HP, LCAT, FBLN1, HPR, IQGAP2, ORM2, SERPINA4, ACTB, IGHG3, TTR, APOH, TOM1, GC, IGKV3-11, CTSD, B2M, CAMP, FGB, AMBP, AHSG, IST1, YWHAZ, GP5, ACTA2, EML5, AZGP1, RBP4, RAB34, COL6A1, ALB, CDH11, SAA1, TAGLN2, SERPINI1, PLCB1, EZR, ATP6V0D1	3.9	5.10E-15
collagen-containing extracellular matrix	15	FGB, SPARC, AMBP, AHSG, SERPINC1, MMP2, PZP, MST1, FBLN1, ORM2, AZGP1, APOH, COL4A3, COL6A1, CTSD	8	4.90E-09
specific granule lumen	7	ARG1, HP, HPR, CTSD, B2M, ORM2, CAMP	22.9	5.80E-07
tertiary granule lumen	6	HP, PPBP, HPR, CTSD, B2M, CAMP	22.1	7.30E-06
platelet alpha granule lumen	6	FGB, SPARC, AHSG, ALB, PPBP, ORM2	18.2	1.90E-05
endocytic vesicle lumen	4	SPARC, SAA1, HP, HPR	41.2	1.20E-04
endoplasmic reticulum lumen	9	RCN1, PROC, F10, AHSG, SERPINC1, COL4A3, ALB, COL6A1, B2M	5.8	1.50E-04
vesicle	6	RAB34, TAGLN2, EZR, YWHAZ, CAMP, ACTB	7.5	1.20E-03
basement membrane	5	ACTA2, SPARC, COL4A3, COL6A1, FBLN1	10.6	1.20E-03
platelet alpha granule	3	FGB, SPARC, PPBP	36.3	3.00E-03
high-density lipoprotein particle	3	APOH, SAA1, LCAT	23.8	6.90E-03
azurophil granule lumen	4	TTR, IST1, ARG1, ORM2	9.1	9.70E-03
actin cytoskeleton	6	ACTA2, TAGLN2, ADCY8, IQGAP2, EZR, ACTB	4.2	1.30E-02
lamellipodium	5	ACTA2, DDX3X, CARMIL3, IQGAP2, ACTB	5.4	1.40E-02

secretory granule lumen	4	DDX3X, AHSG, DBH, SERPINI1	7	1.90E-02
endoplasmic reticulum	12	FGB, RCN1, PROC, AMBP, F10, COL4A3, ALB, POMT1, ACSL5, ATP10A, DBH, B2M	2	3.10E-02
cytosol	37	USP13, HBS1L, TRIM52, SPEF2, DDX3X, CHPF, NGLY1, PBXIP1, IQGAP2, CHTF18, ACTB, PPP3R2, CASP5, METTL4, TOM1, MFN2, BBS9, GC, CTSD, B2M, OFD1, PPP1R14A, AMBP, FANCM, IST1, ARG1, SAMD4B, YWHAZ, ATG13, ACTA2, GOLPH3, REST, GOLGB1, TAGLN2, TTLL11, PLCB1, EZR	1.3	3.80E-02
protein-containing complex	8	ACTA2, FEN1, CFHR1, ALB, COL6A1, PLCB1, EZR, ACTB	2.4	4.90E-02
serine-type endopeptidase activity	9	PROC, F10, C1R, MMP2, MST1, HP, MMP19, KLK15, HPR	9	6.70E-06
antioxidant activity	4	AMBP, ALB, HP, HPR	36.7	1.60E-04
serine-type endopeptidase inhibitor activity	5	AMBP, SERPINC1, PZP, SERPINI1, SERPINA4	8.8	2.50E-03
cadherin binding	6	DDX3X, IST1, CDH11, TAGLN2, EZR, YWHAZ	3.6	2.40E-02
extracellular matrix structural constituent	4	FGB, SPARC, COL4A3, FBLN1	6.4	2.50E-02
hemoglobin binding	2	HP, HPR	77	2.50E-02
protein binding	80	HBS1L, FEN1, SPARC, DHRS11, DDX3X, CHPF, NGLY1, POMT1, HP, WDR41, DBH, CHTF18, ORM2, SERPINA4, ACTB, HERC3, CASP5, C1QTNF6, TOM1, BBS9, B2M, CTSD, CAMP, OFD1, FGB, HEMK1, AHSG, IST1, ARG1, MMP2, MST1, ACSL5, ADRA2C, ATG13, YWHAZ, TENT5D, ACTA2, AZGP1, RBP4, RCN1, RAB34, COL4A3, COL6A1, TAGLN2, EZR, ATP6V0D1, PLCB1, CCDC150, USP13, EPHA10, C1R, SERPINC1, LCAT, FBLN1, ATP10A, PBXIP1, PDHB, PPP3R2, TTR, TAF1B, NSD1, APOH, MFN2, ZNF624, HOXC9, ZNF467, ANKRD26, AMBP, FANCM, F10, SAMD4B, KLK15, PPBP, GP5, GOLPH3, REST, PROC, CFHR1, GOLGB1, ALB	1.1	3.30E-02
calcium ion binding	9	RCN1, SPARC, PROC, PPP3R2, F10, C1R, CDH11, FBLN1, PLCB1	2.3	3.70E-02
identical protein binding	16	OFD1, IST1, C1R, SERPINC1, FBLN1, YWHAZ, ACTB, REST, TTR, CFHR1, C1QTNF6, APOH, ALB, PLCB1, EZR, B2M	1.7	3.80E-02
cysteine-type endopeptidase activity	3	USP13, CASP5, CTSD	8.6	4.70E-02
serine-type peptidase activity	3	PROC, C1R, KLK15	8.5	4.80E-02

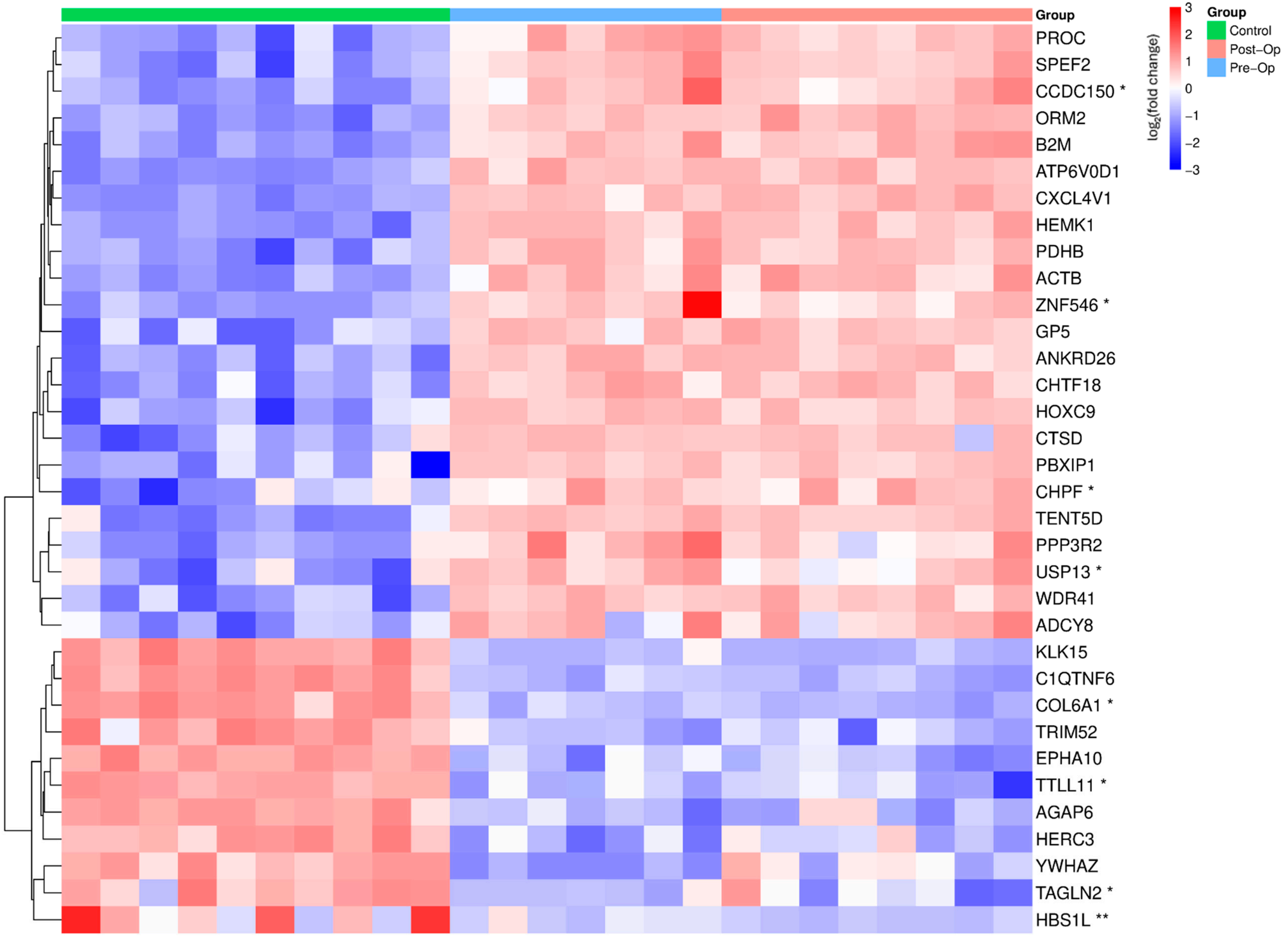
Group
PROC
SPEF2
CCDC150 *
ORM2
B2M
ATP6V0D1
CXCL4V1
HEMK1
PDHB
ACTB
ZNF546 *
GP5
ANKRD26
CHTF18
HOXC9
CTSD
PBXIP1
CHPF *
TENT5D
PPP3R2
USP13 *
WDR41
ADCY8
KLK15
C1QTNF6
COL6A1 *
TRIM52
EPHA10
TTLL11 *
AGAP6
HERC3
YWHAZ
TAGLN2 *
HBS1L **
log2(fold change)
3
2
1
0
−1
−2
−3
Group
Control
Post−Op
Pre−Op

Supplementary Figure S1. Heatmap of five-fold differential proteins across Control, Pre-Op and Post-Op. Displayed are 34 proteins meeting the five-fold criterion ($|\log_2 FC| \geq 2.32$) in at least one comparison with corresponding statistical significance ($p < 0.05$). Among these, 26 proteins satisfy both fold-change and statistical significance criteria at both time points, 7 proteins meet the fold-change threshold in only one comparison (marked with *), and 1 protein (**) exceeds the fold-change threshold at both time points but reaches statistical significance only in the Post-Op comparison. Cells show $\log_2 FC$ values relative to healthy controls, color-mapped around 0 (red = higher, blue = lower). Rows (proteins) are hierarchically clustered to emphasize co-varying patterns; columns (samples) are annotated by group in the top bar (green = Control, blue = Pre-Op, orange = Post-Op).
